# Investigating the impact of meteorological parameters on daily soil temperature changes using machine learning models

**DOI:** 10.1038/s41598-025-04605-0

**Published:** 2025-06-06

**Authors:** Farrokh Asadzadeh, Somayeh Emami, Ahmed Elbeltagi, Muhammed Ernur Akiner, Vahid Rezaverdinejad, Farshid Taran, Ali Salem

**Affiliations:** 1 Department of Soil and Water Conservation, Urmia University, Urmia, 5756151818 Iran; 2 Department of Water Engineering, University of Tabriz, Tabriz, Iran; 3 Agricultural Engineering Department, Mansoura University, Mansoura, 35516 Egypt; 4 Vocational School of Technical Sciences, Department of Environmental Protection Technologies, Akdeniz University, Antalya, Turkey; 5 Department of Water Engineering, Urmia University, Urmia, Iran; 6 Agricultural Research, Education and Extension Organization, Agricultural Engineering Research Institute, P.O. Box 31585‑845, Karaj, Alborz Iran; 7 Civil Engineering Department, Faculty of Engineering, Minia University, Minia, 61111 Egypt; 8 Structural Diagnostics and Analysis Research Group, Faculty of Engineering and Information Technology, University of Pécs, Pécs, 7622 Hungary

**Keywords:** Artificial neural network (ANN), Soil temperature (ST), Seasonal ARIMA (SARIMA), Meteorological parameters, Predictive modeling, Soil depth, Climate sciences, Environmental sciences, Solid Earth sciences

## Abstract

Soil temperature (ST) is one of the critical parameters in agricultural meteorology and significantly influences physical, chemical, and biological activities in the soil environment. One of the major challenges in agricultural studies is the limited number of synoptic stations for measuring ST. Novel data mining methods offer effective solutions for obtaining reliable estimations while reducing costs and improving accuracy. This study estimated daily ST at depths of 5 cm, 10 cm, 20 cm, 50 cm, and 100 cm using meteorological parameters from an American synoptic station over three years (2020–2022). Seasonal ARIMA (SARIMA), Multiple Linear Regression (MLR), and Artificial Neural Network (ANN) models were applied for ST prediction. A bivariate correlation test with a p-value less than 0.05 was conducted to determine the relationship between meteorological variables and ST. The results indicate that the ANN model outperforms SARIMA and MLR in predicting ST at all depths. For instance, at 5 cm depth, the ANN model achieved RMSE = 0.85, *r* = 0.98, MAE = 1, and PBIAS = 1.5%, compared to SARIMA (RMSE = 1.5, *r* = 0.96, MAE = 1.16, PBIAS = 2.5%) and MLR (RMSE = 1.35, *r* = 0.97, MAE = 1.3, PBIAS = 3%). Similarly, at 100 cm depth, the ANN model achieved RMSE = 0.65, *r* = 0.91, MAE = 0.55, and PBIAS = 2.2%, compared to SARIMA (RMSE = 1.3, *r* = 0.96, MAE = 0.58, PBIAS = 3.5%) and MLR (RMSE = 1.15, *r* = 0.91, MAE = 0.68, PBIAS = 3.8%). The analysis also revealed that surface temperature (Avg. Infrared) and air temperature (Avg. T) were the most influential parameters in ST prediction. Additionally, the ANN model exhibited the lowest error rates across all depths, highlighting its superior capability for estimating daily ST in agricultural soils. This study provides a comprehensive framework for accurately estimating daily ST at different depths, offering valuable insights for agricultural and environmental applications.

## Introduction

Soil temperature and its temporal-spatial changes are some of the most critical factors that directly or indirectly depend on the amount and direction of soil physical processes. This research addresses a crucial challenge in agricultural meteorology: accurately and efficiently predicting daily soil temperature (ST) at various depths using limited synoptic station data. The primary aim of this study is to evaluate and compare the performance of three predictive modeling approaches—Seasonal ARIMA (SARIMA), Multiple Linear Regression (MLR), and Artificial Neural Networks (ANN)—to establish a cost-effective and scalable solution for ST estimation. By leveraging data from Riley Station in Oregon, the study provides a reliable framework for addressing gaps in ST measurement, a pressing issue in sustainable agricultural management. Importantly, this work emphasizes how these models can mitigate challenges arising from the high costs and logistical difficulties associated with direct ST measurements.

### Research gaps

Soil temperature (ST) is critical in many areas, such as agricultural production, soil microbiology, plant growth, and climate change^1^. However, accurate measurement and prediction of soil temperature pose a significant challenge, mainly due to the limited number of synoptic stations and high measurement costs^2^. Various statistical and machine learning models have been proposed to predict soil temperature. For example, models such as Multiple Linear Regression (MLR), Seasonal ARIMA (SARIMA), and Artificial Neural Networks (ANN) have been used to predict soil temperature at different depths^3^. However, the performance of these models has generally been limited to specific depths or regional conditions^4^. Furthermore, the number of comprehensive studies in which these models have been comparatively evaluated at different soil depths and under various meteorological conditions is quite limited^5^. This creates a significant research gap in the generalizability and applicability of the models used in soil temperature prediction.

### Purpose of the study

The primary purpose of this study is to compare the performances of Seasonal ARIMA (SARIMA), Multiple Linear Regression (MLR), and Artificial Neural Networks (ANN) models to predict daily soil temperature at different soil depths (5 cm, 10 cm, 20 cm, 50 cm, and 100 cm) and to determine the most suitable model. For this purpose, models were developed and tested using three years (2020–2022) of meteorological data from Riley Station in Oregon. The study aims to deeply examine the role of meteorological parameters, especially surface temperature (Avg. Infrared) and air temperature (Avg. T), in soil temperature prediction^6,7^. In addition, this study aims to provide more accurate and reliable prediction models for agricultural and environmental applications by comparing the performance of models used in soil temperature prediction at different depths^8^. This way, a more cost-effective and scalable solution proposal will be presented for predicting soil temperature in regions with limited synoptic stations.

### Literature review

Soil temperature is affected by several factors, including topography, solar radiation, air temperature, precipitation distribution, soil moisture level, soil type, and thermal properties, such as heat capacity, thermal conductivity coefficient, and specific heat^9^. Soil temperature plays a significant role in critical processes and surface energy balance as a storage source in the atmosphere^10^.

Since the ST regime directly affects crop growth, its diversity, and the biological activities of the soil, the reconstruction of the statistical deficiency of ST is of high importance in bioclimatic and agricultural studies^11^. Different sensors or thermometers are needed to determine soil temperature changes at various depths. Meteorological data above the soil surface is one of the most valuable methods of predicting soil temperature^9^. Statistical and experimental methods are suitable for estimating ST in points without measurements^12,13^.

The parameters of maximum soil temperature and depth of glaciers are not considered indirect measurements in a short period^2^. Mathematical relationships and suitable simulation methods to estimate the annual changes in soil temperature in each region meet the needs of industrial and construction projects^1^. The researchers conducted ST modeling and analysis are classified into theoretical and practical categories^14,15^. In theoretical studies, the influential factors in ST changes include incoming short-wavelength radiation, heat flux, soil texture, outgoing long-wavelength radiation, soil moisture. These factors are measured with advanced and accurate measuring equipment^16^. Theoretical models are limited to laboratory studies due to the need for data and various unmeasured input variables^17^. In applied studies, changes in ST are estimated using existing meteorological variables and different calculation methods^2^. In addition, data mining methods are used for soil temperature modeling. Machine learning and artificial intelligence methods are more important than classical statistical methods in modeling due to more accurate results^4,18^.

Multivariable linear regression, genetic algorithm, neuro-fuzzy system, neuro-fuzzy neural network, and artificial neural network have higher accuracy in estimating parameters related to ST prediction^19^. Several studies have been conducted to assess soil temperature from meteorological parameters using linear or non-linear methods, models based on machine learning, and time series^5,13,20^. Nahvi et al.^21^ used a self-adaptive evolutionary algorithm (SaE) to improve the performance of extreme learning machines (ELM). The results showed that the ELM model accurately estimates soil temperature. Zare Abyaneh et al.^22^ estimated soil temperature at different depths using an artificial neural network (ANN) and active neural-fuzzy inference system (CANFIS). Comparing the performance of the models showed that ANN capabilities are more favorable than CANFIS in predicting ST. Citakoglu^18^ used artificial neural networks (ANN), adaptive neural-fuzzy inference system (ANFIS), and multiple linear regression (MLR) models to predict soil temperature at different depths. The results showed that the ANFIS model provides more optimal results than ANN and MLR models. Araghi et al.^14^ predicted the soil temperature at different depths using artificial neural network ANN and wavelet artificial neural network (WANN). The results show a good agreement between the measured and estimated values. Samadianfard et al.^23^ predicted the soil temperature at different depths with the firefly algorithm and found that the actual values ​​of the soil temperature differed slightly from the expected values.

Feng et al.^3^ used fast machine learning models (ELM), generalized regression neural networks (GRNN), back propagation neural networks (BPNN), and random forests (RF). The ELM model has better performance and calculation speed. Wang et al.^24^ proposed a new model based on a one-dimensional convolutional neural network-multilayer perceptron (1D-CNN-MLP) for predicting soil temperature. Li et al.^7^ designed the long short-term memory (LSTM) model to predict soil moisture (SM) and ST. The results show that the proposed ILSTM- Soil model is more optimal than random forest (RF), support vector regression (SVR), and elastic network (ENET). Malik et al.^25^ predicted daily soil temperature at different depths using hybrid machine learning models and found that the SVM-SMA model provides more favorable estimates than other models in different soil depths. Farhangmehr et al.^26^ used a 1-dimensional CNN model to predict hourly soil temperature at various soil depths and concluded that a 1-dimensional CNN model performs better than a multilayer perceptron in predicting soil temperature. Zhou et al.^27^ evaluated the performance of CMIP6 models in simulating ST. Using observation data, grid data (TS-GCB), and reanalysis data (ERA5L), the accuracy of these models was checked for surface soil (0–5 cm) and subsurface soil (5–15 cm). The results showed that the multi-model ensemble mean (MME) generally captures soil temperature changes, but overestimates the simulated temperature (1.86 °C for the surface and 2.16 °C for the subsurface). Also, the highest simulation error was observed in tropical regions, and positive and negative fluctuations were observed in dry regions. Wang et al.^28^ estimated ST based on 2-meter air temperature (T2) using ST-U-Net deep learning model. The model was trained in the Lushan Mountains region and obtained an average absolute error of less than 0.8 K. Adding environmental variables reduced the error by 26.7%, and a physical training strategy reduced it by 8.8%. This study showed the high accuracy of deep learning in predicting ST. Malik et al.^29^ used four machine learning models (RF, RBNN, MLPNN, and CANFIS) to predict daily ST at 5, 15, and 30 cm depths at Bathinda station, Punjab (India) during 2016–2019. The temperature data (Tmean), relative humidity (RH), wind speed (WS), and sunshine duration (SSH) were evaluated as input. The CANFIS model provided the most accurate results (MAE = 0.636–0.806 °C, RMSE = 0.854–1.074 °C, PCC = 0.993–0.995), indicating its high efficiency in ST estimation using meteorological data.

### The innovative aspects

The soil temperature parameter dramatically affects the growth and activity of soil microorganisms and crop growth. The innovative aspects of the study are as follows:

- A comprehensive analysis to predict daily soil temperature at different depths is a significant contribution, especially for agricultural management and climate change adaptation.
- SARIMA, MLR, and ANN models were compared, and their performances at different soil depths were evaluated. This provides a comprehensive approach that examines the depth-based performances of the models, unlike similar studies in the literature.
- Analyzing the effect of meteorological parameters such as surface and air temperature on soil temperature at different depths provides a deeper understanding of soil temperature prediction.
- This study extends previous studies on soil temperature prediction and provides a new methodological framework to understand and predict soil temperature dynamics at different depths, which can be considered an essential step, especially for agricultural management and climate change adaptation.
- This study contributes a robust, data-driven framework that bridges theoretical advancements and practical applications, addressing the gaps in predictive accuracy and field adaptability observed in previous works.

## Materials and methods

### Case study

**Fig. 1:**
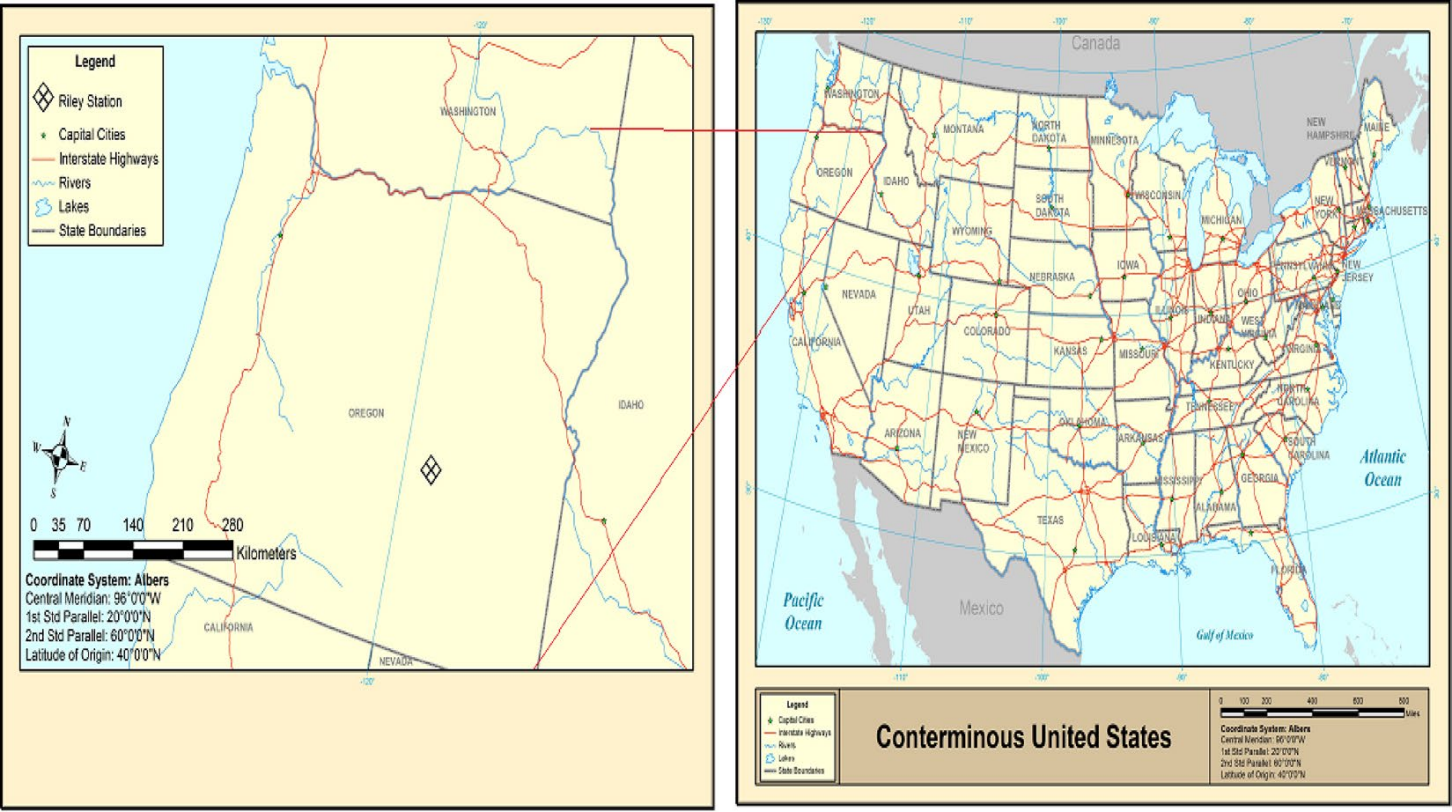
A description of the study area’s location.

Riley supplied the dataset that was used. It is situated in the United States in Harney County, Oregon, at the intersection of U.S. S. Riley Station’s daily ST data over an extended period was used. Riley Station was discovered at 1288 m above sea level, at 43°32′30′′ N and 119°30′14′′ W. The average annual temperature and precipitation were 10.6 °C and 279 mm. The location of the study area is depicted in Fig. 1.

The calculated daily values of 2020 and 2021 were used for analytical measurement. The total number of data is 870. The samples from 2020 to 2021 (*n* = 728) were used to train the models, and the 2022 (*n* = 142) samples were used for validation. A bivariate correlation test with a P-value less than 0.05 was performed.

Meteorological variables and daily ST were measured, as shown in Table 1.

**Table 1:** Meteorological-related variables and associated sensors.

Air temperature:	Three platinum resistance thermometers housed in fan-aspirated solar radiation shields.
Precipitation:	An inlet-heated, wind-shielded weighing rain gauge (configured with three load cell sensors), precipitation (wetness) detector, and an auxiliary tipping bucket gauge.
Wind speed:	A 3-cup anemometer at the same height as the air temperature shield intakes.
Solar radiation:	A silicon pyranometer.
Surface (skin): temperature.	A precision infrared temperature sensor pointed at the ground surface
Relative humidity:	A capacitive thin-film polymer humidity sensor provides accurate and stable measurement even in high-humid environments.
Soil temperature & moisture:	Moisture sensors with built-in thermistors installed at specific depths: 5, 10, 20, 50, and 100 cm.

## Methodology

**Fig. 2:**
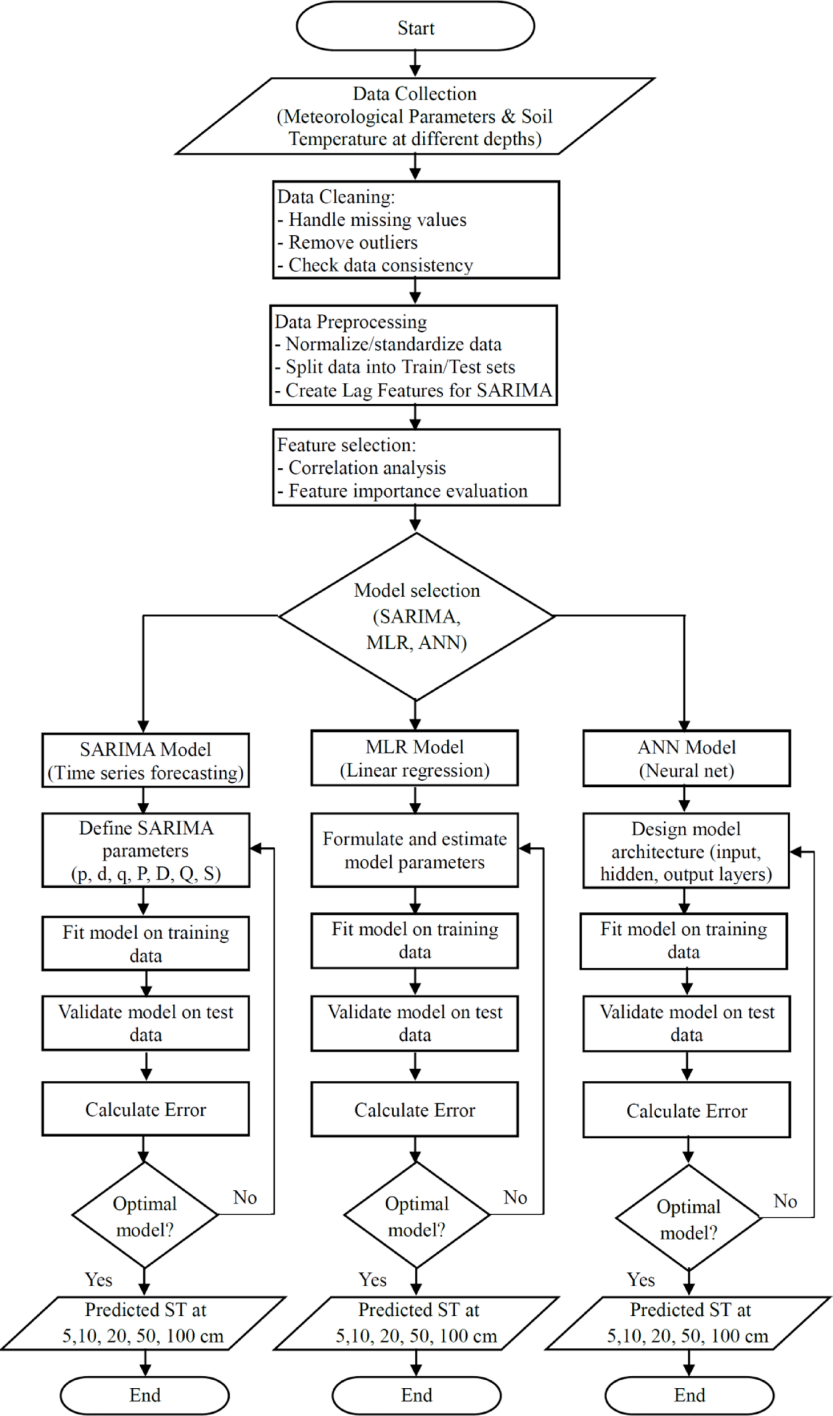
The flowchart for the structured overview of the research workflow.

In this study, models for seasonal ARIMA (SARIMA), multiple linear regression (MLR), and artificial neural networks (ANN) were applied. The SARIMA model was applied to forecast soil temperature (ST) time series 2022. The MLR model was built using a traditional statistical technique. Finally, the appropriate ANN model architecture was chosen. Figure 2 presents the workflows of the SARIMA, MLR, and ANN models in a single, integrated flow chart. The process begins with Data Collection and Preprocessing, where meteorological data and soil temperature measurements are gathered and prepared for analysis.

The main reasons behind the selection of Seasonal ARIMA (SARIMA), Multiple Linear Regression (MLR) and Artificial Neural Networks (ANN) models in the study are as follows:

1. Filling the Gap in the Literature: Many studies on soil temperature estimation usually focus on a single model or a specific soil depth. This study aims to fill this gap in the literature by comprehensively comparing SARIMA, MLR and ANN models to estimate daily soil temperature at different soil depths (5 cm, 10 cm, 20 cm, 50 cm and 100 cm).

In particular, studies comparing the performances of these models at different depths are limited. Therefore, this study aims to determine which model is more effective at which depth for soil temperature estimation.

2. Advantages and Suitability of Models: SARIMA (Seasonal ARIMA): It is a highly effective method for modeling seasonal fluctuations and trends in time series data. Seasonal changes significantly affect soil temperature, and SARIMA is a suitable option for modeling such data.
3. MLR (Multiple Linear Regression): MLR is a simple and effective method to model linear relationships between independent variables (meteorological parameters) and dependent variable (soil temperature). This model provides a basis for soil temperature prediction and a reference point to compare the performance of other models.
4. ANN (Artificial Neural Networks): ANN successfully models complex and non-linear relationships. Soil temperature is intricately related to meteorological parameters, and ANN is an ideal option for capturing such relationships. Moreover, ANN’s deep learning capacity allows us better to understand the temperature dynamics at different soil depths.
5. Suitability for Practical Applications: SARIMA, MLR and ANN models are widely used in agricultural and environmental applications such as soil temperature prediction. These models provide reliable results from both theoretical and practical perspectives.

ANN, in particular, is well suited for problems such as soil temperature estimation due to its ability to process large data sets and model complex relationships.

6. Comparative Analysis Opportunity: This study aims to determine which model performs better under which conditions by comparing different models (statistical and machine learning models). This serves as a guide for researchers and practitioners on which model to use and when.

### Seasonal ARIMA (SARIMA)

The SARIMA model is one of the general linear models for seasonal time series forecasting^30^. The general form of the model is SARIMA*(p*,* d*,*q*)×(*P*,* D*,* Q*)W. The model’s second part considers the time series’ seasonal part. The SARIMA model form is as follows:

1 $${Q_P}=\left( {{B^\omega }} \right){\phi _p}\left( B \right)\nabla _{\omega }^{D}{z_t}={\theta _Q}\left( {{B^\omega }} \right){\theta _q}\left( B \right){\varepsilon _t}$$.

where *p* indicates the order of the non-seasonal autocorrelation model, *q* indicates the order of the non-seasonal moving average model, *P* indicates the order of the seasonal autocorrelation model, *Q* suggests the order of the seasonal moving average model, *B* indicates the difference operator, $\nabla _{\omega }^{D}$ means the D-mean Seasonal difference as *W*, *Φ* is the parameter of the non-seasonal autocorrelation model, and *θ* is the parameter of the non-seasonal moving average model. The time series modeling process is based on the initial analysis of the time series, determination of model ranks, parameter estimation, and model goodness of fit test^31^. The pseudo code of the SARIMA model is shown in Table 2.

**Table 2:** Pseudo code for SARIMA model.

# SARIMA model
# Step 1: Load and preprocess data
Load dataset from Riley Station (2020–2021 for training, 2022 for validation)
Preprocess data (handle missing values, normalize if necessary)
# Step 2: Define SARIMA model parameters
*p* = 3 # Non-seasonal autoregressive order
d = 1 # Non-seasonal differencing order
q = 4 # Non-seasonal moving average order
*P* = 0 # Seasonal autoregressive order
D = 1 # Seasonal differencing order
Q = 1 # Seasonal moving average order
s = 365 # Seasonal period (daily data with yearly seasonality)
# Step 3: Fit SARIMA model
SARIMA_model = SARIMA(train_data, order=(p, d, q), seasonal_order=(P, D, Q, s))
SARIMA_model_fit = SARIMA_model.fit()
# Step 4: Validate SARIMA model
predictions = SARIMA_model_fit.predict(start = validation_start_date, end = validation_end_date)
Calculate RMSE, r, and residuals for validation data.
# Step 5: Evaluate model performance
Plot predictions vs. actual values
Generate residual plots
Calculate AIC, BIC, and other statistical metrics.
# Step 6: Forecast future soil temperature
future_predictions = SARIMA_model_fit.forecast(steps = number_of_future_days)

### Multiple linear regression (MLR)

Multiple Linear Regression (MLR) is a statistical method used to model the relationship between a dependent variable and two or more independent variables. This study applied MLR to predict daily soil temperature (ST) at various depths by leveraging meteorological parameters as predictors. The model’s foundation lies in analyzing how independent variables collectively influence the dependent variable, allowing for the quantification of each variable’s contribution.

The MLR analysis calculated regression coefficients for independent variables, representing their relative contributions to ST prediction. A stepwise regression technique was employed to refine the model and identify key predictors. The model’s performance was evaluated using root mean square error (RMSE), Pearson correlation coefficient (r), and residual plots. These metrics provided robust validation against observed ST values.

**Fig. 3:**
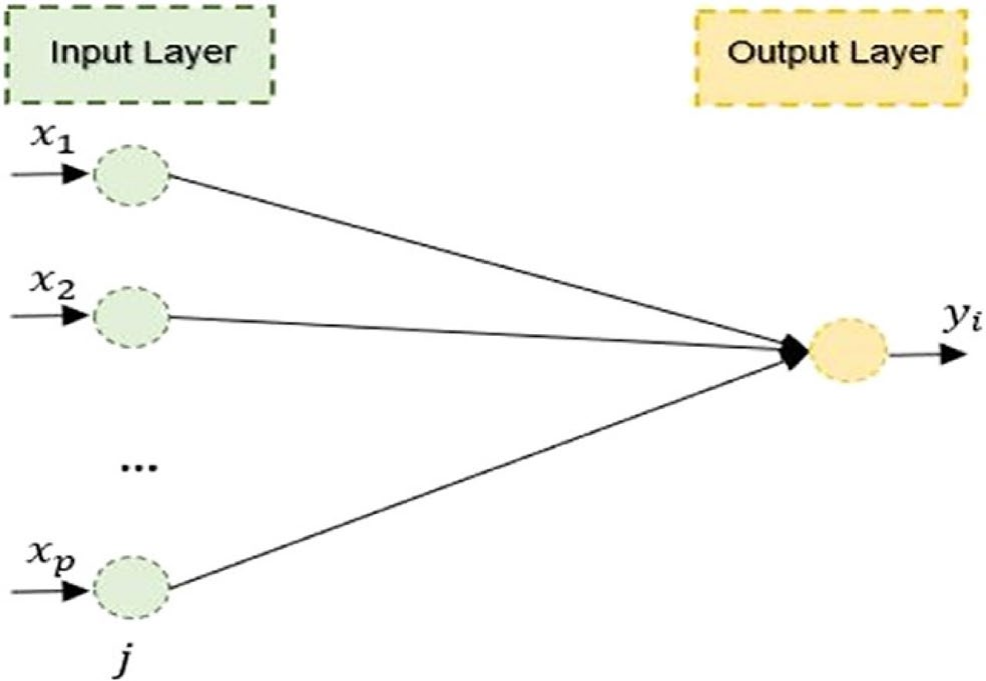
Schematic of the MLR model^21^.

This statistical approach establishes a baseline for comparing the predictive capabilities of more advanced models, such as Artificial Neural Networks (ANN) and Seasonal ARIMA (SARIMA). The general schematic of the MLR model is shown in Fig. 3. The pseudo code of the MLR model is shown in Table 3.

**Table 3:** Pseudo code for MLR model.

# MLR model
# Step 1: Load and preprocess data
Load dataset from Riley Station (2020–2021 for training, 2022 for validation)
Preprocess data (handle missing values, normalize if necessary)
Perform bivariate correlation analysis to select significant predictors (p-value < 0.05)
# Step 2: Define MLR model
MLR_model = LinearRegression()
# Step 3: Fit MLR model
MLR_model.fit(train_data[independent_variables], train_data[dependent_variable])
# Step 4: Validate MLR model
predictions = MLR_model.predict(validation_data[independent_variables])
Calculate RMSE, r, and residuals for validation data.
# Step 5: Evaluate model performance
Plot predictions vs. actual values
Generate residual plots
Calculate regression coefficients and R-squared values.
# Step 6: Predict future soil temperature
future_predictions = MLR_model.predict(future_data[independent_variables])

### Artificial neural networks (ANN)

Artificial Neural Networks (ANNs) are computational models inspired by the structure and functioning of the human brain^32^. These models mimic the brain’s ability to learn from data by employing self-adaptive learning algorithms. The complexity of ANNs arises from their multilayer structure, consisting of an input layer, one or more hidden layers, and an output layer, with nodes (neurons) in each layer interconnected to process and transfer data^42,43^. The ANN’s intricate network of weighted connections enables it to detect complex, non-linear relationships among variables.

Unlike the MLR model, which assumes linearity, ANNs leverage activation functions to introduce nonlinearity, capturing intricate interactions between meteorological variables and ST. The design of an ANN involves five fundamental steps^32^: (1) Gathering Data, (2) Preprocessing Data, (3) Network Building, (4) Training, and (5) Modeling Test Performance. These steps ensure that the model is optimized for its specific task. The pseudo code of the ANN model is shown in Table 4.

**Table 4:** Pseudo code for ANN model.

# ANN model
# Step 1: Load and preprocess data
Load dataset from Riley Station (2020–2021 for training, 2022 for validation)
Preprocess data (handle missing values, normalize data)
Split data into training (80%) and validation (20%) sets
# Step 2: Define ANN architecture
input_layer_size = number_of_input_features
hidden_layer_1_size = 10
hidden_layer_2_size = 10
output_layer_size = 1 # Predicting soil temperature at a specific depth
# Step 3: Initialize ANN model
ANN_model = Sequential()
ANN_model.add(Dense(hidden_layer_1_size, input_dim = input_layer_size, activation=’relu’))
ANN_model.add(Dense(hidden_layer_2_size, activation=’relu’))
ANN_model.add(Dense(output_layer_size, activation=’linear’))
# Step 4: Compile ANN model
ANN_model.compile(optimizer=’adam’, loss=’mean_squared_error’, metrics=[‘accuracy’])
# Step 5: Train ANN model
ANN_model.fit(train_data[independent_variables], train_data[dependent_variable], epochs = 100, batch_size = 32)
# Step 6: Validate ANN model
predictions = ANN_model.predict(validation_data[independent_variables])
Calculate RMSE, r, and residuals for validation data.
# Step 7: Evaluate model performance
Plot predictions vs. actual values
Generate residual plots
Calculate correlation coefficient and RMSE.
# Step 8: Predict future soil temperature
future_predictions = ANN_model.predict(future_data[independent_variables])

**Fig. 4:**
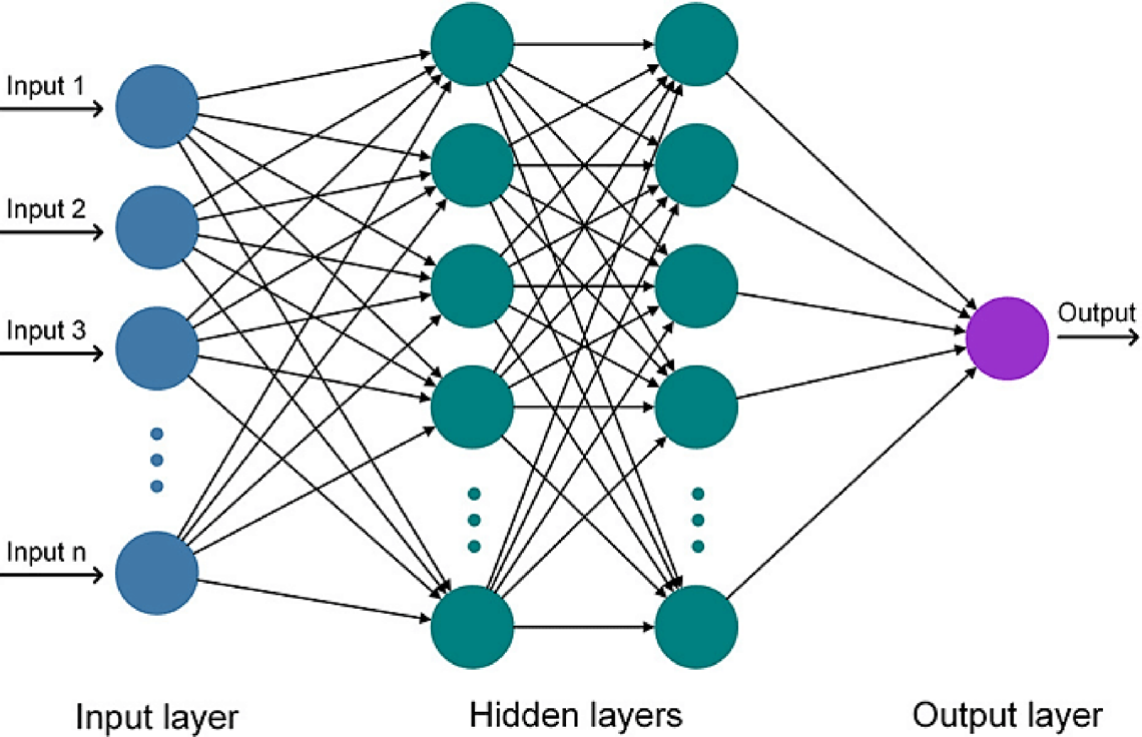
The ANN model’s schematic^20,34–36^.

This research demonstrates the ANN’s superior capability to handle non-linear dependencies and interactions compared to MLR and SARIMA models. By leveraging its ability to capture complex temporal and spatial variations, the ANN model is positioned as a robust tool for ST prediction. Figure 4 illustrates the general schematic of the ANN model.

Moreover, the ANN architecture reflects the computational parallels of the human brain, where countless neurons interact to produce results^37^. This complexity underscores the advantages of ANN in handling multifaceted, non-linear processes, such as those required for ST prediction.

The determination of the ANN structure was based on a combination of factors, including^39^:

1. Training and validation performance: The performance of the ANN was evaluated using a validation set, ensuring the model’s ability to generalize to unseen data. Models with too few or too many nodes were rejected based on their ability to minimize RMSE and maximize the Pearson correlation coefficient (r).
2. Computational efficiency: The complexity of the model (i.e., the number of nodes and layers) was adjusted to balance accuracy and processing time.
3. Overfitting/underfitting considerations: By adjusting the number of nodes and layers, the model was optimized to avoid overfitting (too many nodes) or underfitting (too few nodes) the data.

This structure was implemented using Waikato Environment for Knowledge Analysis (WEKA) software Version 3.8.6 (https://ml.cms.waikato.ac.nz/weka), and the model’s configuration was further refined through iterative testing to ensure robustness in predicting ST at various depths. The final ANN model, with two hidden layers of 10 nodes each, provided the best overall performance, as evidenced by its validation against actual ST measurements.

### Validation of the models

A bivariate correlation analysis guided the selection of variables for the MLR model, ensuring that only statistically significant variables (p-value < 0.05) were included. The null hypothesis was tested for each variable to determine its correlation with ST, with statistical significance defined by p-values ranging from 0.01 to 0.05. Variables such as average air temperature (Avg. T) and surface temperature (Avg. Infrared) emerged as influential predictors, while precipitation was excluded due to its negligible impact on ST.

This study used the ANN model to simulate ST at various depths (5, 10, 20, 50, and 100 cm). WEKA software was employed to implement the ANN, making this the first application of these models for daily ST simulation in the study area. Data preprocessing involved normalizing the input variables and splitting the dataset into training (80%) and validation (20%) subsets. The backpropagation algorithm was utilized for training, iteratively adjusting weights to minimize prediction errors. The ANN’s performance was validated using RMSE, r, and residual plots, ensuring robust calibration and generalization to unseen data.

In this study, the architecture of the Artificial Neural Network (ANN) model was carefully designed to optimize its predictive performance for daily soil temperature (ST) estimation. The ANN model consists of three layers: an input layer, two hidden layers, and an output layer. The input layer contains the meteorological variables, such as air temperature, surface temperature, and humidity, which serve as the model’s input data. The output layer produces the predicted daily soil temperature for each depth.

For the hidden layers, the model was constructed with two layers, each containing 10 nodes. The choice of the number of nodes and layers was determined using a trial-and-error approach based on the performance metrics (RMSE, r). The number of hidden layers and nodes was fine-tuned to achieve the best performance during the training process. This approach was validated by testing various configurations to determine the optimal balance between computational efficiency and predictive accuracy. In this case, two hidden layers with 10 nodes each provided the most reliable and consistent results, with minimal overfitting or underfitting.

The comparison of SARIMA, MLR, and ANN models is based on the Pearson correlation coefficient (r), the root mean squared error (RMSE), the residual plots, and Percentile Bias (PBIAS), which are the most common reliable metrics for determining the statistical models’ data-based performance (Eq. 2 to 5).

The correlation coefficient measures the linear relationship between the predictions and the actual values and is used to evaluate the model’s predictive ability^33^. The correlation coefficient is calculated as follows:

2 $$r=\frac{{\mathop \sum \nolimits_{{i=1}}^{N} \left( {{y_i} - {}^\backprime y} \right)\left( {{{\hat {y}}_i} - {}^\backprime \hat {y}} \right)}}{{\sqrt {\mathop \sum \nolimits_{{i=1}}^{N} {{({y_i} - {}^\backprime y)}^2}\mathop \sum \nolimits_{{i=1}}^{N} {{({{\hat {y}}_i} - {}^\backprime \hat {y})}^2}} }}~~~$$.

RMSE is the root mean square of the prediction error and is a widely used metric to evaluate model performance^37,38^. RMSE is calculated as follows:

3 $$RMSE=\sqrt {\frac{1}{N}\mathop \sum \limits_{{i=1}}^{N} {{({y_i} - {{\hat {y}}_i})}^2}}$$.

Residual analysis is used to evaluate the distribution of errors in model predictions and helps identify systematic errors of the model^42^. Residuals are calculated as follows:

4 $$\operatorname{Re} sidual={y_i} - {\hat {y}_i}$$.

Standard deviation is a measure of the distance of values in a data set from the mean. It shows the spread and variability of the data.

Percentile Bias (PBIAS) is a statistical measure that measures the tendency of a model’s predictions to be overestimated or underestimated compared to observed values^40^. A positive PBIAS indicates that the model is underestimated, while a negative PBIAS demonstrates that it is overestimated. It is widely used in hydrological and environmental modeling to assess prediction accuracy.

5 $$PBIAS\left( \% \right)=\frac{{\sum \left( {{y_i} - {{\hat {y}}_i}} \right)}}{{\sum {y_i}}} \times 100$$.

Where $\:{y}_{i}$ is the observed values, $\:{\widehat{\text{y}}}_{\text{i}}$ is the predicted values, $\:N$ is the number of observations. A PBIAS value close to zero indicates an unbiased model, while larger deviations indicate systematic over- or underestimation.

## Results and discussion

The findings of this study reinforce and extend the conclusions of prior research in soil temperature modeling. For instance, Citakoglu^18^ demonstrated that ANFIS and ANN models outperform classical statistical methods for ST estimation. Building on this, our results show that the ANN model achieved a superior correlation coefficient (*r* = 0.98) and reduced RMSE values, particularly at depths of 50 cm and 100 cm, compared to SARIMA and MLR models. Similarly, while Feng et al.^3^ identified ELM as the most effective method, our ANN approach demonstrates higher adaptability and accuracy under the unique meteorological conditions of Riley Station.

This study also highlights the influence of meteorological parameters, with surface temperature (Avg. Infrared) and air temperature (Avg. T) identified as the most critical variables for accurate ST prediction. These findings align with Sattari et al.^6^, emphasizing air temperature’s importance in their predictive models. Significantly, our work extends these insights by quantitatively demonstrating how the ANN model outperforms other machine-learning techniques in capturing non-linear and depth-dependent variations in ST.

These results validate the applicability of ANN models for complex ST prediction tasks and highlight their practical benefits in agricultural management. The ANN model’s ability to accurately predict ST with minimal input parameters suggests a transformative potential for field applications, enabling cost and time efficiency. By advancing the understanding of model performance across depths and conditions, this research establishes a benchmark for future studies in soil temperature modeling.

### Modeling results

Identifying whether the data has a specific correlation due to seasonal fluctuations is critical. For this reason, input data for 2020 and 2021 were categorized into hot and cold seasons, and then correlation analysis was done independently for each season. To investigate the distribution characteristics of ST at different depths, descriptive statistical indices such as mean, standard deviation, minimum, maximum, skewness and kurtosis were calculated (Table 5).

**Table 5:** Descriptive statistical indices of soil temperature at different depths.

Soil depth (cm)	Mean	Standard deviation (Std)	Min.	Max.	Skewness	Kurtosis
5	0.172	0.106	0.00	0.516	0.028	−1.098
10	0.191	0.093	0.013	0.371	−0.217	−1.220
20	0.296	0.075	0.099	0.484	−0.494	−0.587
50	0.271	0.094	0.061	0.549	0.045	−0.290
100	0.308	0.098	0.083	0.446	−0.748	−0.927

The results show that the average ST has a relatively stable trend with increasing depth, but the value of the standard deviation at some depths shows changes that indicate temperature fluctuations in these layers. Skewness values are close to zero in most depths, which indicates a relatively symmetrical distribution of data. However, at the depths of 10, 20 and 100 cm, negative skewness is observed, which indicates the skewness of the data distribution towards values lower than the mean. On the other hand, Kurtosis values are negative in all depths, which indicates that the data distribution is wider and has shorter tails than the normal distribution. These patterns indicate the relative stability of temperature in different soil depths as well as the possible influence of environmental and seasonal conditions on temperature variability.

**Fig. 5:**
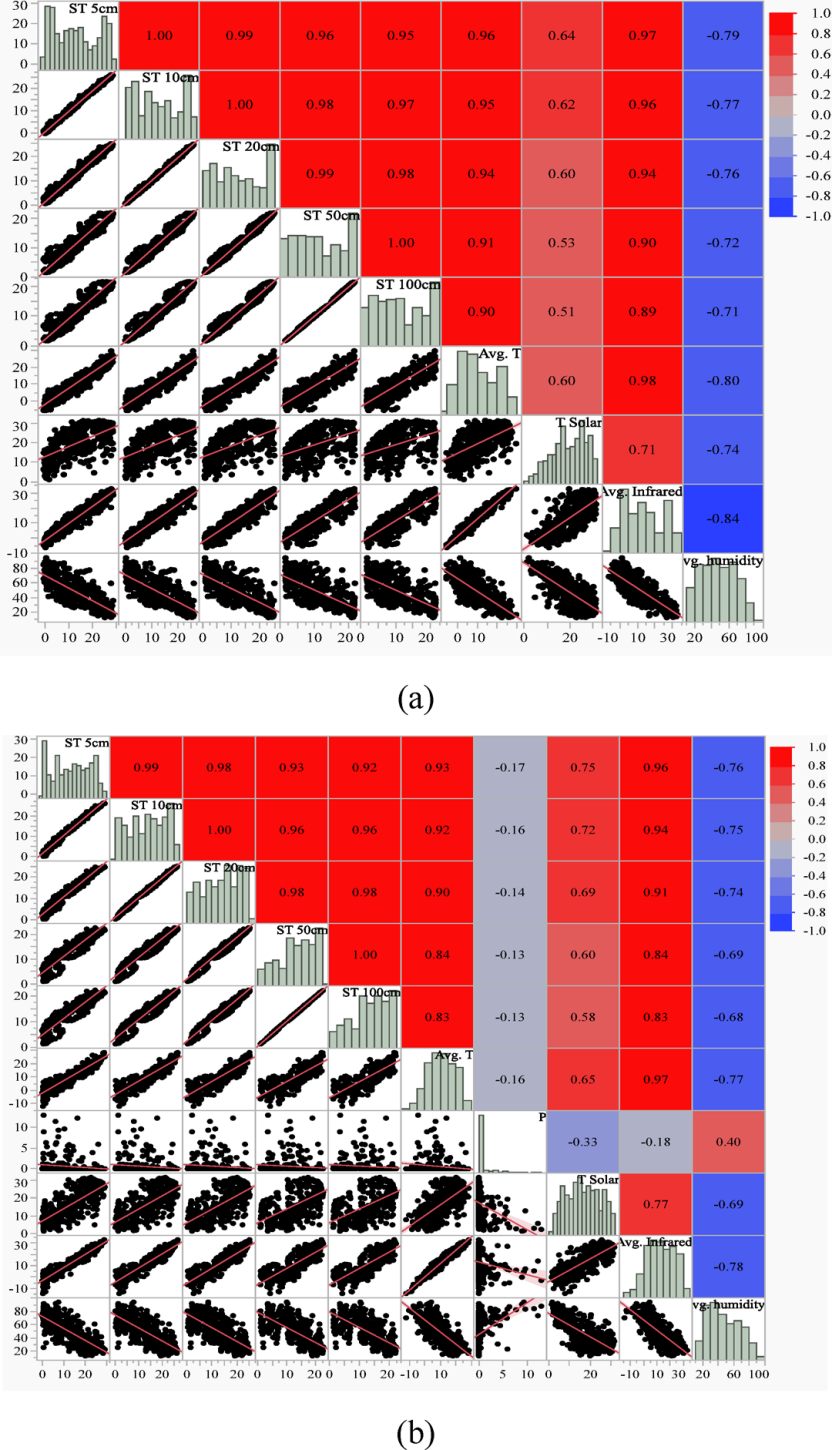

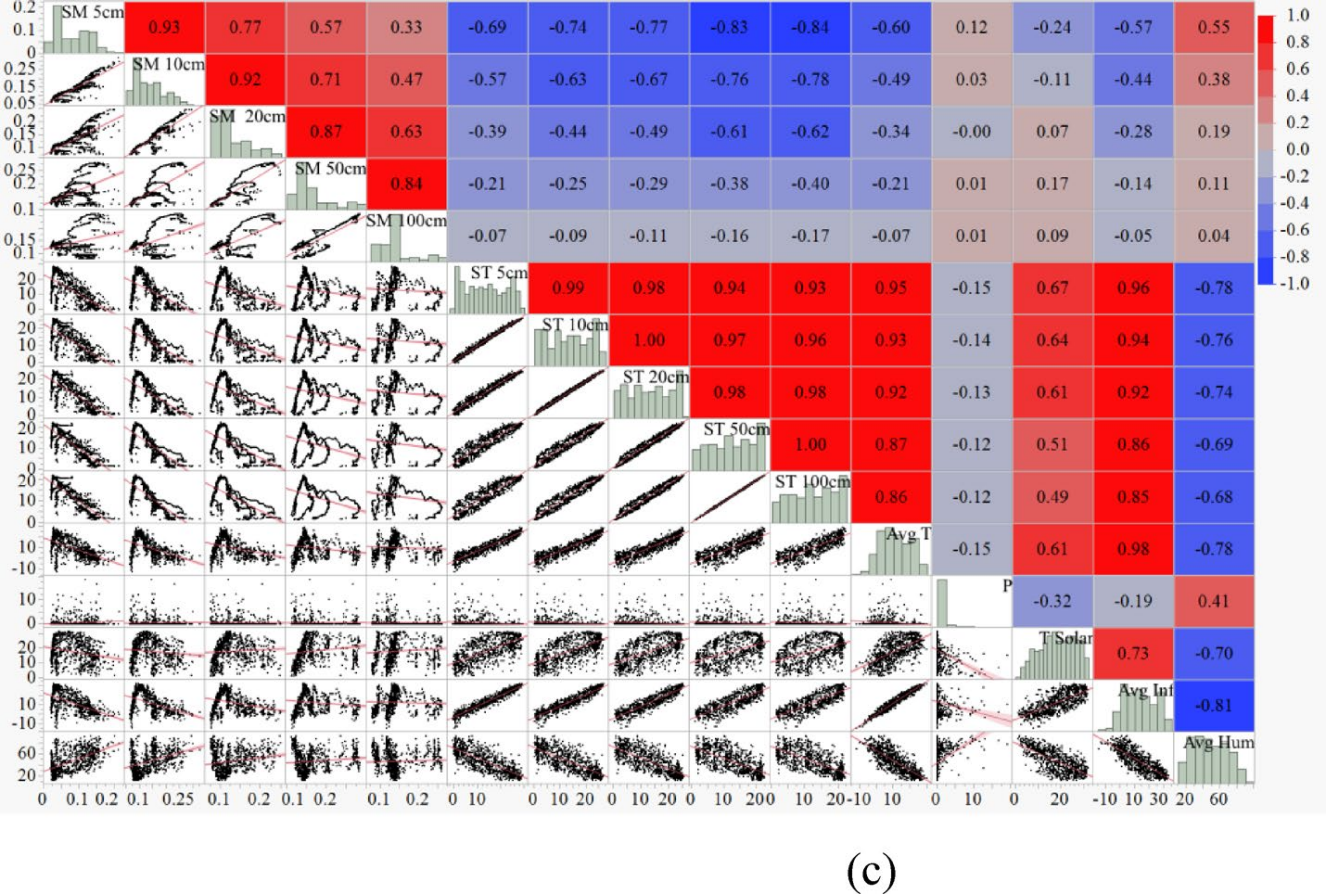
Correlation coefficient scatterplots of soil temperature (ST) at various depths (5, 10, 20, 50, and 100 cm) with meteorological parameters: average temperature (Avg. T), precipitation (P), solar radiation (T Solar), surface temperature (Avg. Infrared), and humidity (Avg. Humidity). The plots represent data for (**a**) hot seasons, (**b**) cold seasons, and (**c**) the combined daily dataset from 2020 to 2021.

Figure 5 (a to c) shows the scatterplot matrix for two seasons, i.e., hot and cold. The matrix comprehensively shows the correlations between dependent and independent variables. Data from January, February, March, October, November, and December were used for the cold season. For the hot season, data from the other months, April, May, June, July, August, and September, were utilized as input in the correlation study. The results reveal that seasonal fluctuation has little influence on soil temperature at various depths Fig. 5 (a and b). As a result, it was determined that separating the data would be ineffective. As a result, the association between the parameters was discovered utilizing the whole daily data set for 2020 and 2021. Figure 5 (c) shows independent factors and correlation values that greatly influence daily ST. When measuring soil temperatures at depths of 5, 10, and 20, it was found that the surface (skin) temperature (Avg. Infrared) was the most significant meteorological variable, while air temperature (Avg. T) was substantial at depths of 50 and 100 cm.

The analysis revealed that the influence of meteorological parameters on ST varies significantly with depth. At shallower depths of 5, 10, and 20 cm, the surface (skin) temperature, represented by the average infrared (Avg. Infrared) parameter, emerged as the most significant variable. This can be attributed to the direct and immediate effect of surface radiation and heat transfer on the upper soil layers, where thermal energy from the surface penetrates without substantial attenuation. As a result, fluctuations in surface temperature are strongly correlated with ST at these depths.

In contrast, at deeper soil layers (50 and 100 cm), air temperature (Avg. T) became the dominant influencing factor. This shift is likely due to the diminished direct impact of surface radiation as thermal energy dissipates while moving downward through the soil. At these depths, heat transfer processes, such as conduction, rely more on the ambient air temperature, which exerts a more consistent and long-term influence. These findings align with the principles of thermal conductivity in soils, where heat transfer at greater depths is slower and primarily influenced by stable external factors like air temperature.

The distinct differences in the significant predictors across soil depths highlight the importance of considering depth-specific meteorological parameters in soil temperature modeling. This insight also reinforces the need for tailored predictive models that account for varying physical and thermal dynamics at different soil layers.

Based on the analyses, SARIMA was deemed appropriate for the forward prediction of daily ST data with the indicated seasonality pattern. The SARIMA model results are given in Table 6. The SARIMA model was built using daily data from 2020 to 2021. The model’s effectiveness was evaluated using data collected in 2022.

**Table 6:** Model summary for the seasonal ARIMA (3, 1, 4) (0, 1, 1).

	ST 5 cm	ST 10 cm	ST 20 cm	ST 50 cm	ST 100 cm
DF	430	430	430	430	430
Sum of squared ınnovations	796.4261	358.441824	202.834375	36.619936	29.0858254
Sum of squared residuals	1031.4257	406.50709	222.668526	43.621358	37.8301503
Variance estimate	1.8521537	0.83358564	0.47170785	0.0853612	0.06779913
Standard deviation	1.3609386	0.91300911	0.68680991	0.2921663	0.26038265
Akaike’s ‘A’ ınformation criterion	1638.5095	1232.53964	970.887492	265.25938	205.517234
Schwarz’s Bayesian criterion	1675.27	1269.30014	1007.64799	301.99935	242.257204
RSquare	0.9676638	0.98488737	0.99047255	0.9974801	0.99767799
RSquare Adj	0.9670622	0.9846062	0.9902953	0.9974331	0.99763469
MAPE	–	–	10.522491	4.0397258	3.37647498
MAE	1.1663581	0.73294892	0.5499343	0.223755	0.19560323
−2LogLikelihood	1620.5095	1214.53964	952.887492	247.25938	187.517234
Stable	Yes	Yes	Yes	Yes	Yes
Invertible	Yes	Yes	Yes	Yes	Yes

**Fig. 6:**
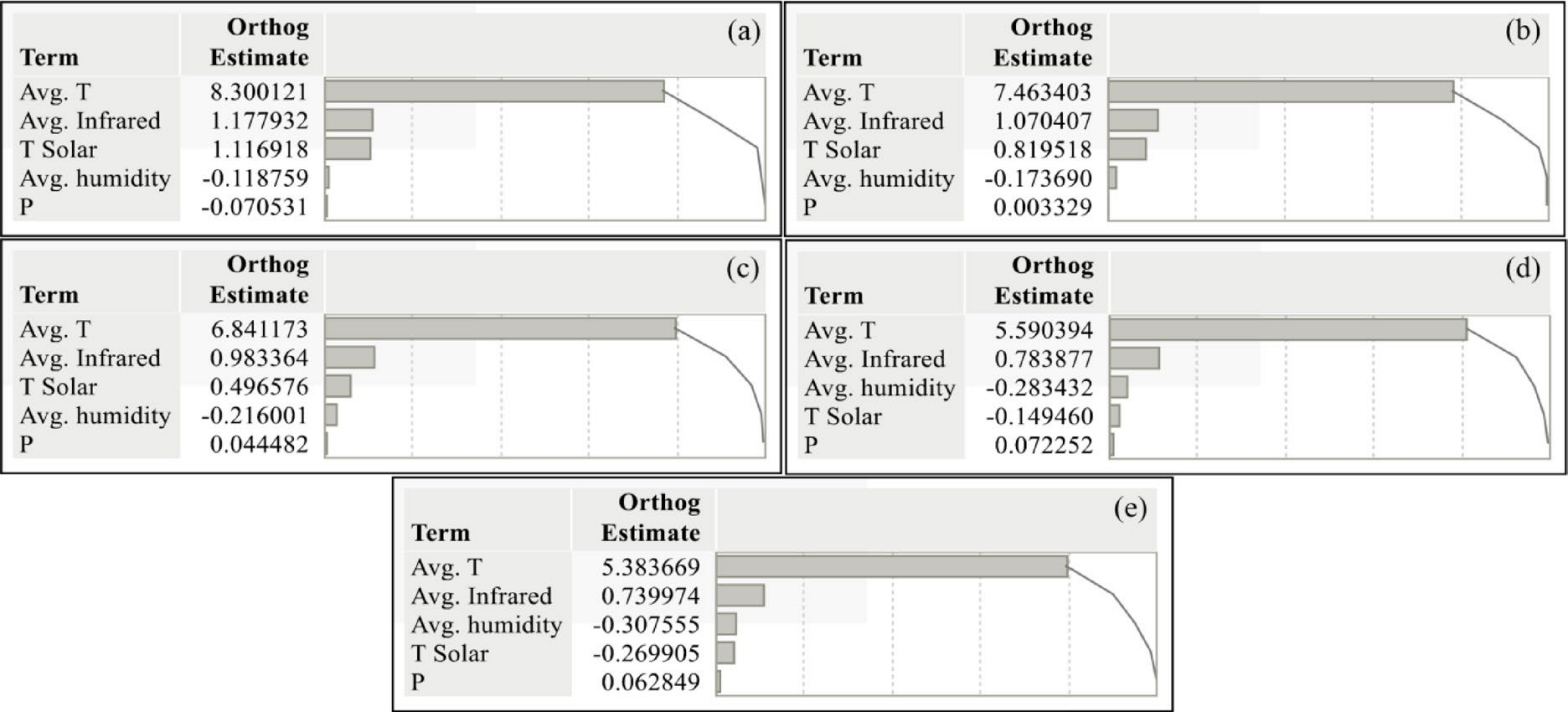
(**a**–**e**) Pareto plot of orthogonal estimates (**a**) ST 5 cm, (**b**) ST 10 cm, (**c**) ST 20 cm, (**d**) ST 50 cm, (**e**) ST 100 cm.

The orthogonal least squares estimation approach was used to investigate the importance of input variables in predicting daily ST at different depths^15^. An absolute value Pareto plot of the estimates is included in the Fit Last Squares Pareto plot report^40^. The order of model term entry affects the orthogonal estimates. Estimates are transformed into orthogonal forms and standardized for equal variances. The importance of input variables in predicting daily ST for different depths is presented in Tables 7, 8, 9, 10 and 11; Fig. 6 (a-e). Average air temperature (Avg. T), solar radiation (T Solar), surface temperature (Avg. Infrared), and relative humidity (Avg. Humidity) are the essential meteorological variables in daily ST prediction.

In comparison, precipitation (P) has the most negligible significance on daily ST. In addition, the effect of precipitation on soil temperature is insignificant in all models and different soil depths. As a result, the precipitation parameter was ignored in the used models.

In Fig. 6 (a-e), Pareto charts showing the importance of meteorological parameters used in estimating daily soil temperature at different soil depths (5 cm, 10 cm, 20 cm, 50 cm and 100 cm) are presented, and these charts were constructed using the orthogonal least squares method in order to determine the essential meteorological parameters for each depth; in the charts, the effect of the parameters on the estimation is expressed by the “Orthog t-Ratio” values ​​and the parameters with the highest values stand out as the most important factors; while the surface temperature (Avg. Infrared) is the most critical parameter at the depths of 5 cm, 10 cm and 20 cm, the air temperature (Avg. T) plays a more decisive role at the depths of 50 cm and 100 cm; also the effect of other parameters such as solar radiation (T Solar) and humidity (Avg. Humidity) remains at lower levels, while precipitation (P) is observed to be the parameter with the least effect at all depths.

**Table 7:** Parameter estimate population through least squares fit (ST 5 cm).

Term	Estimate	t ratio	Orthog coded	Orthog t-ratio	Prob>|t|
Intercept	4.18326	6.3725	13.2525	166.2184	< 0.0001*
Avg. T	−0.04387	−0.7057	8.3001	104.1034	< 0.0001*
P	0.11983	2.4536	−0.0705	−0.8846	0.3766
T solar	−0.08160	−3.4976	1.1169	14.0088	< 0.0001*
Avg. ınfrared	0.87936	14.8050	1.1779	14.7741	< 0.0001*
Avg. humidity	−0.01184	−1.4895	−0.1188	−1.4895	0.1367

**Table 8:** Parameter estimate population through least squares fit (ST 10 cm).

Term	Estimate	t ratio	Orthog coded	Orthog t-ratio	Prob>|t|
Intercept	5.91412	8.1427	13.1607	149.1905	< 0.0001*
Avg. T	−0.04070	−0.5917	7.4634	84.6056	< 0.0001*
P	0.12930	2.3930	0.0033	0.0377	0.9699
T solar	−0.11275	−4.3677	0.8195	9.2901	< 0.0001*
Avg. ınfrared	0.80024	12.1771	1.0704	12.1342	< 0.0001*
Avg. humidity	−0.01732	−1.9690	−0.1737	−1.9690	0.0493*

**Table 9:** Parameter estimate population through least squares fit (ST 20 cm).

Term	Estimate	t ratio	Orthog coded	Orthog t-ratio	Prob>|t|
Intercept	7.41377	9.5152	13.0706	138.1201	< 0.0001*
Avg. T	−0.02464	−0.3340	6.8412	72.2924	< 0.0001*
P	0.11311	1.9513	0.0445	0.4701	0.6384
T solar	−0.15190	−5.4852	0.4966	5.2474	< 0.0001*
Avg. ınfrared	0.73616	10.4422	0.9834	10.3915	< 0.0001*
Avg. humidity	−0.02154	−2.2825	−0.2160	−2.2825	0.0227*

**Table 10:** Parameter estimate population through least squares fit (ST 50 cm).

Term	Estimate	t ratio	Orthog coded	Orthog t-ratio	Prob>|t|
Intercept	9.79549	11.3110	12.4232	118.1124	< 0.0001*
Avg. T	0.02745	0.3348	5.5904	53.1501	< 0.0001*
P	0.04838	0.7508	0.0723	0.6869	0.4923
T solar	−0.22321	−7.2518	−0.1495	−1.4210	0.1557
Avg. ınfrared	0.58876	7.5139	0.7839	7.4526	< 0.0001*
Avg. humidity	−0.02827	−2.6947	−0.2834	−2.6947	0.0072*

**Table 11:** Parameter estimate population through least squares fit (ST 100 cm).

Term	Estimate	t ratio	Orthog coded	Orthog t-ratio	Prob>|t|
Intercept	10.3120	11.8288	12.3308	116.4596	< 0.0001*
Avg. T	0.0433	0.5241	5.3837	50.8466	< 0.0001*
P	0.0316	0.4869	0.0628	0.5936	0.5529
T solar	−0.2359	−7.6130	−0.2699	−2.5491	0.0110*
Avg. ınfrared	0.5565	7.0551	0.7400	6.9888	< 0.0001*
Avg. humidity	−0.0307	−2.9047	−0.3076	−2.9047	0.0038*

The results determined that surface temperature (Avg. Infrared) is the most essential meteorological variable in estimating daily ST at 5, 10, and 20 cm depths. In comparison, air temperature (Avg. T) is the most effective meteorological parameter in assessing daily ST at 50 cm and 100 cm depths. In a similar study, Sattari et al.^6^ stated that air temperature is the most critical parameter in predicting ST, which is consistent with the results of the present study. The difference in the importance of parameters is probably related to the complex simulation process and non-linear mathematical relationships between independent and dependent variables in different predicting models^41^.

**Fig. 7:**
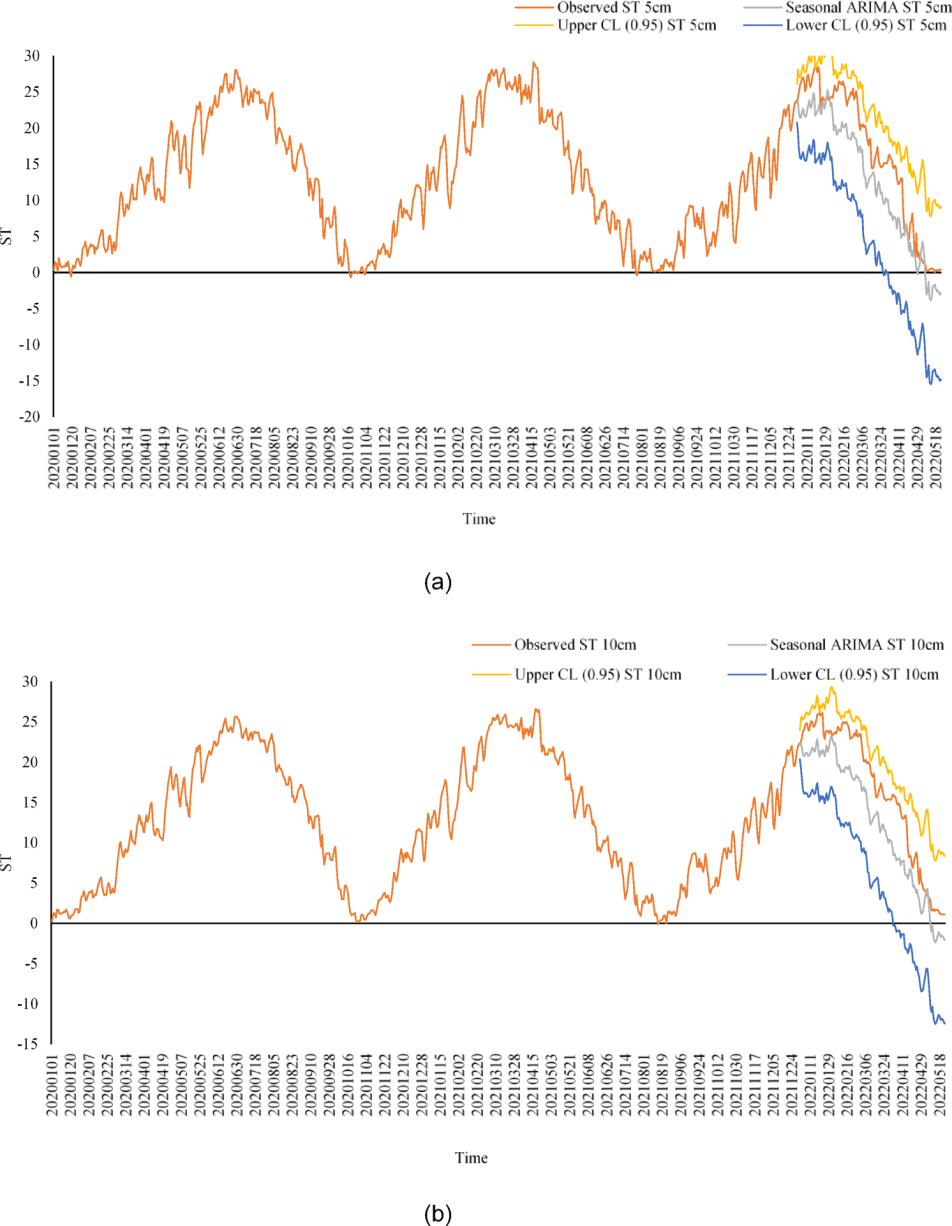

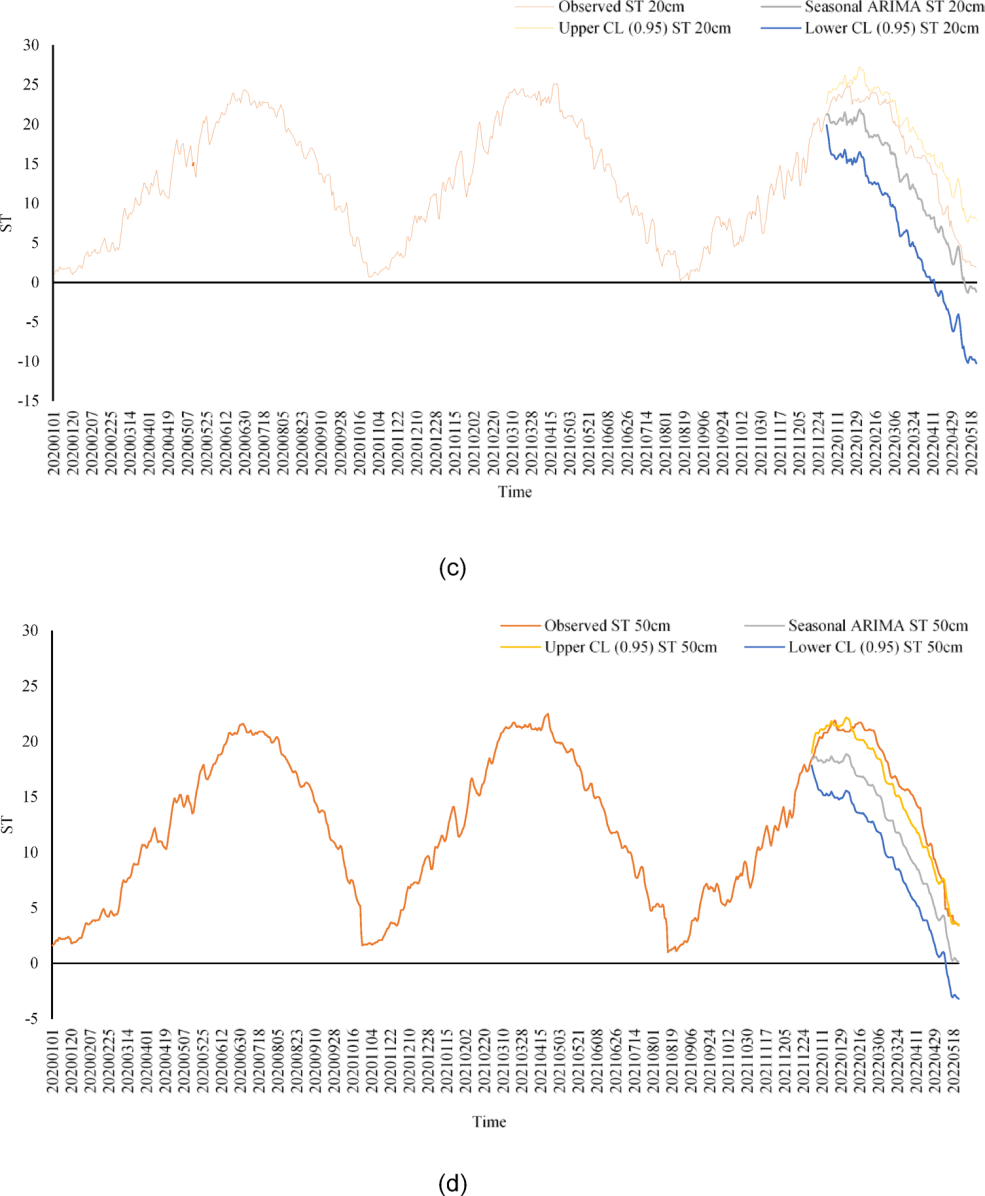

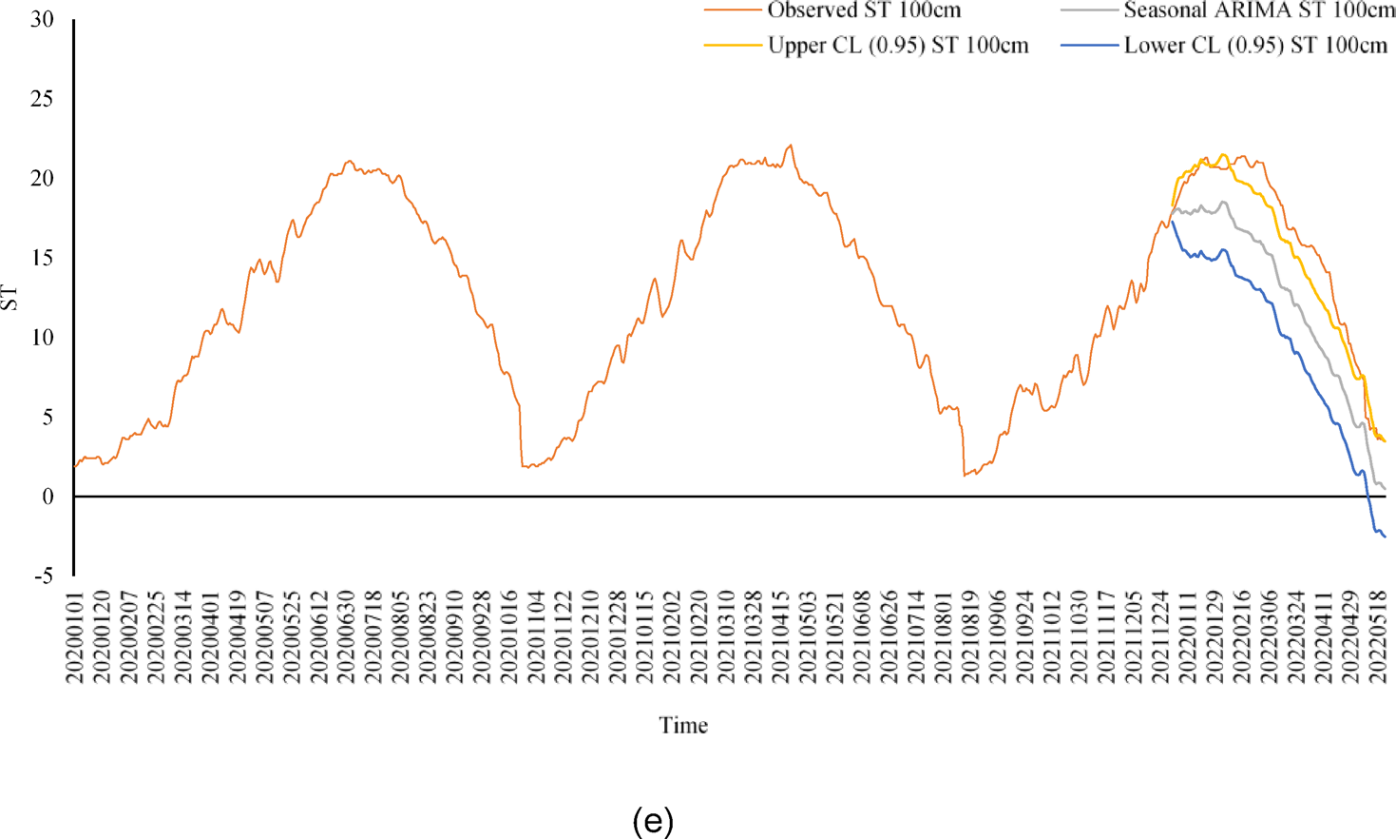
(**a**–**e**) Estimation of ST by SARIMA model for different depths (**a**) 5 cm, (**b**) 10 cm, (**c**) 20 cm, (**d**) 50 cm, and (**e**) 100 cm.

Figure 7 shows the daily soil temperature (ST) estimates of the SARIMA model at different soil depths (5 cm, 10 cm, 20 cm, 50 cm and 100 cm) within a 95% confidence interval. SARIMA captures seasonal fluctuations and trends, especially at near-surface depths (5 cm, 10 cm, 20 cm). A strong agreement is observed between the model’s estimates and the actual values at these depths. However, the model performs relatively poorly at deep soil layers (50 cm, 100 cm). This is because temperature changes in deep soil layers are slower and more complex. Although SARIMA effectively captures seasonal and short-term trends, it may be limited in modeling long-term dynamics in deep soil layers. Therefore, it is recommended that more advanced models (e.g. ANN) be used in deep soil layers. The SARIMA model provides reliable estimates, especially in near-surface soil layers, which provides a significant advantage in agricultural applications. A potent tool is time series forecasting with the most minor inaccuracy. The components are split into systematic and non-systematic components to help pick forecasting methods for time series. Non-systematic components cannot be directly modeled, whereas systematic components are recognizable, regularly repeating components. Trend and seasonality are categorized as systematic components, whereas noise is non-systematic^42^.

**Fig. 8:**
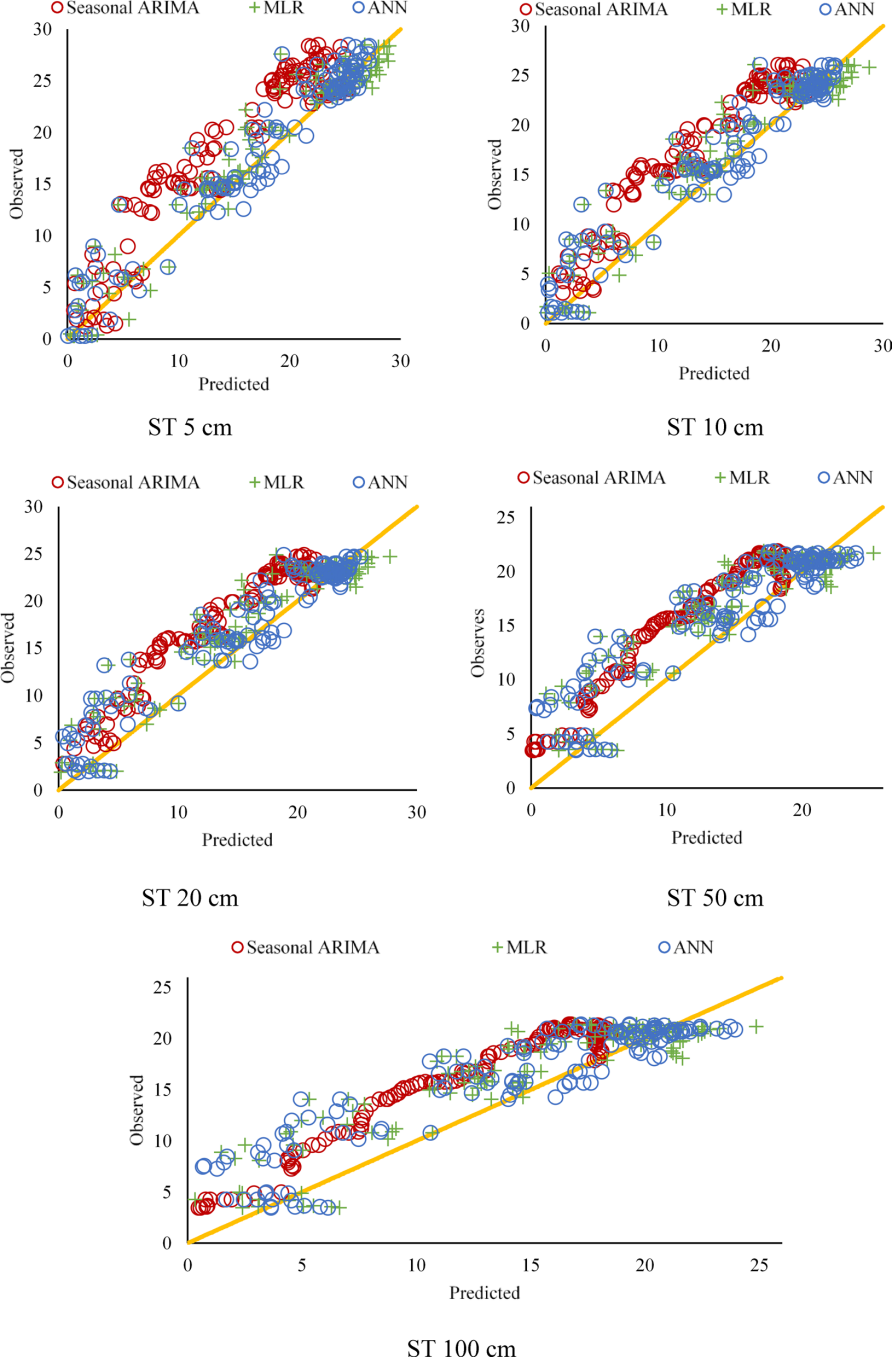
Estimation of ST by SARIMA, MLR, and ANN models for different depths.

**Fig. 9:**
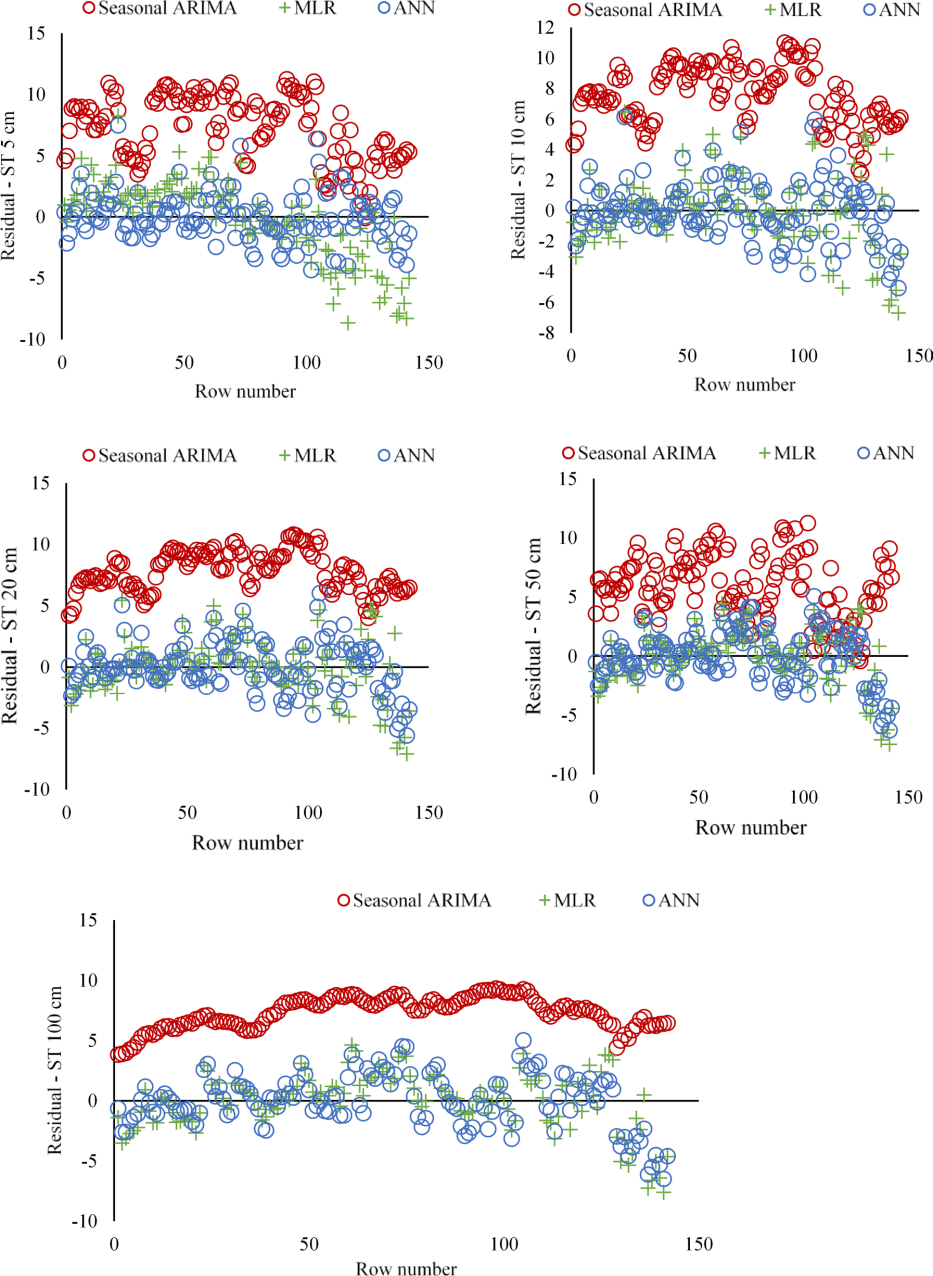
Residual plots for SARIMA, MLR, and ANN models for different depths.

Table 12 displays the models developed using MLR analysis and least square Estimation prediction expressions. In addition, the predicted daily ST values by the models and the observed values are compared in Fig. 8 for different depths. The residual diagrams of the studied models are shown in Fig. 9.

**Table 12:** Least square estimation- MLR prediction expression.

	Parameter estimation				
Term	ST 5 cm	ST 10 cm	ST 20 cm	ST 50 cm	ST 100 cm
Intercept	3.505671	5.1428984	6.5988884	9.0469109	9.6159349
Avg. T	−0.163978	−0.18041	−0.178313	−0.143214	−0.128902
T Solar	−0.115972	−0.152248	−0.19256	−0.263413	−0.275159
Avg. Infrared	1.032646	0.9766981	0.9257365	0.7913915	0.7571906
Avg. humidity	−0.003515	−0.007899	−0.01228	−0.022134	−0.02531

Figure 8 shows that the ANN model performs better than the SARIMA and MLR models at different soil depths. These findings support that the ANN model is the most effective for predicting soil temperature. Furthermore, the graph’s deviations and errors reveal the models’ limitations and areas for improvement.

SARIMA Model: The estimates based on time series analysis successfully captured the seasonal fluctuations, especially at the near-surface depths (5 cm, 10 cm, 20 cm). However, more significant deviations were observed in the estimates at the deep soil layers (50 cm, 100 cm). This is due to the more complex temperature dynamics in the deep layers.

MLR Model: The estimates based on linear regression showed an acceptable performance, especially at the near-surface depths. However, since it could not capture non-linear relationships, the prediction errors increased in deep soil layers. This reveals the limitations of the MLR model.

ANN Model: The artificial neural network model agrees with actual observations at all depths. In particular, its ability to capture complex and non-linear relationships provided more accurate predictions in near-surface and deep soil layers. This proves the superior performance of the ANN model.

Near-Surface Depths (5 cm, 10 cm, 20 cm): All models performed relatively well in near-surface layers. However, the ANN model had the lowest error margin and the highest prediction correlation.

Deep Soil Layers (50 cm, 100 cm): While the prediction performance of the SARIMA and MLR models decreased in deep layers, the ANN model showed a consistent performance. This indicates that ANN can better model the complex temperature dynamics in deep layers.

The comparison of different machine learning algorithms for all soil depths shows the high capability of proposed models in predicting daily ST. The ANN model offers a more reliable statistical relationship based on the calculated statistical indicators than other models.

The regression coefficients express a favorable fit between the observed and the predicted values by the ANN model. The most suitable model fitted in 5 cm to 100 cm soil layers is related to the ANN model. The acceptable performance of the ANN model has also been reported in the study of other researchers, which is consistent with the results of the present study^23,43–45^. The MLR and SARIMA models performed less than the ANN model based on the evaluation criteria values. However, MLR and SARIMA models have acceptable predictions of daily ST, which can help estimate daily ST in the impossibility of using the ANN model. According to Khan et al.^46^, ANNs are a fresh subset of soft computing with a wide range of applications. It is, therefore, possible to categorize or forecast future values using the ANN model.

The performance of neural network models is not required in simple operations; the goal is to use them in models requiring intensive calculations. It has been effectively used in numerous fields because of its fault tolerance. It is applied in fields including categorization, image recognition and optimization, handwriting recognition, churn analysis, meteorological forecast, and soil property prediction to achieve high application performance^46,47^. Artificial neural networks (ANNs) can detect complex temporal variations^46^. For rapid, low-data requirements, ANN offers the advantage of a data-driven, functional, and user-friendly approach^48^. The results of predicting models show that daily ST estimation based on meteorological data decreases with increasing soil depth. The effect of soil moisture on heat transfer in the soil is one of the main reasons for this problem. Several studies have reached similar results, which agree with the present study’s results^18,23,49,50^. Data mining models perform better monthly ST estimation at 5 and 10-cm soil depths^3,8,51^. Yusefi et al.^52^ similarly found that ST ​​decreased with increasing depth, and the correlation between ST and meteorological parameters decreased.

**Fig. 10:**
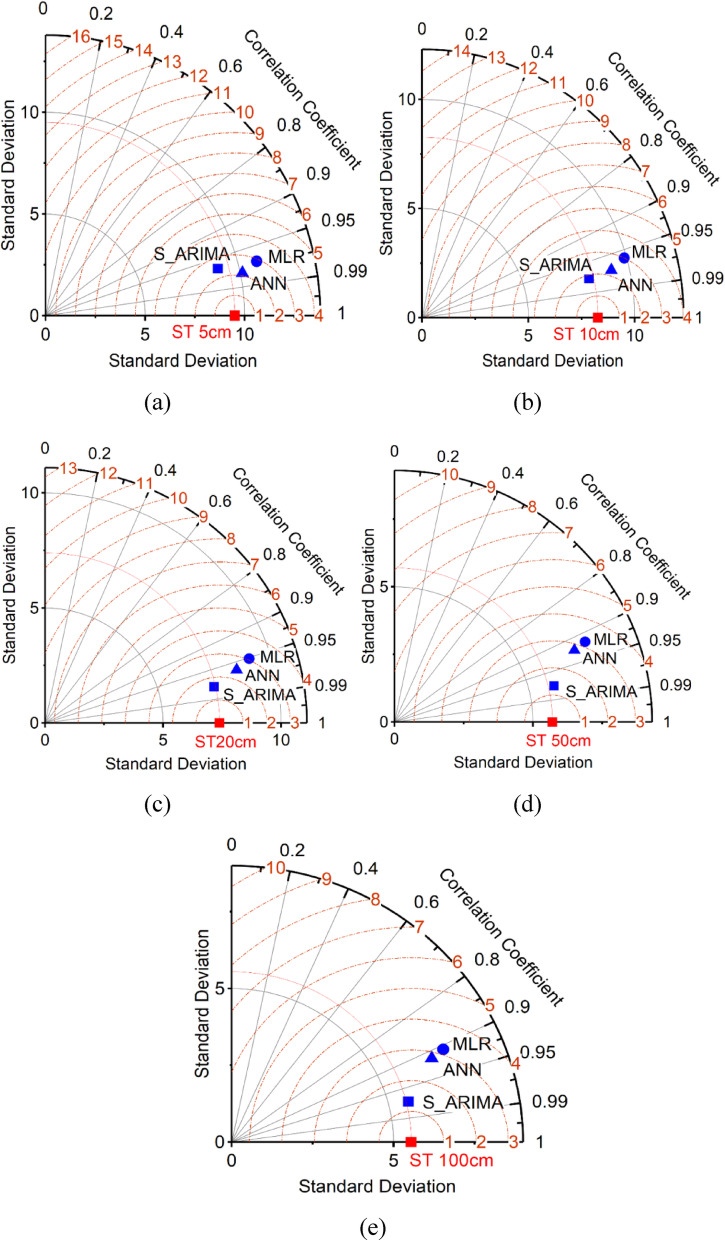
(**a**–**e**) Taylor diagrams of the SARIMA, MLR, and ANN models for different depths (**a**) 5 cm; (**b**) 10 cm; (**c**) 20 cm; (**d**) 50 cm; (**e**) 100 cm.

Figure 10 clearly shows that the ANN model is superior to other models in soil temperature prediction. Taylor diagrams are an effective tool for visually comparing the performance of models, and in this study, the ANN model stands out with higher accuracy and lower error rates. These findings support the idea that the ANN model can be a reliable tool for soil temperature prediction in agricultural and environmental applications. The results show that the ANN model with *r* = 0.98 outperforms the SARIMA and MLR models in forecasting future daily ST at different depths. The highest and lowest determination coefficients between daily ST and meteorological parameters are related to 5–20 cm depths. The results showed that these coefficients decrease with increasing soil depth. The results of the studies of Tabari et al.^5^ and Citakoglu^18^ are based on the accuracy of ANN in predicting ST compared to other data mining methods, which agrees with the results of the present study.

**Fig. 11:**
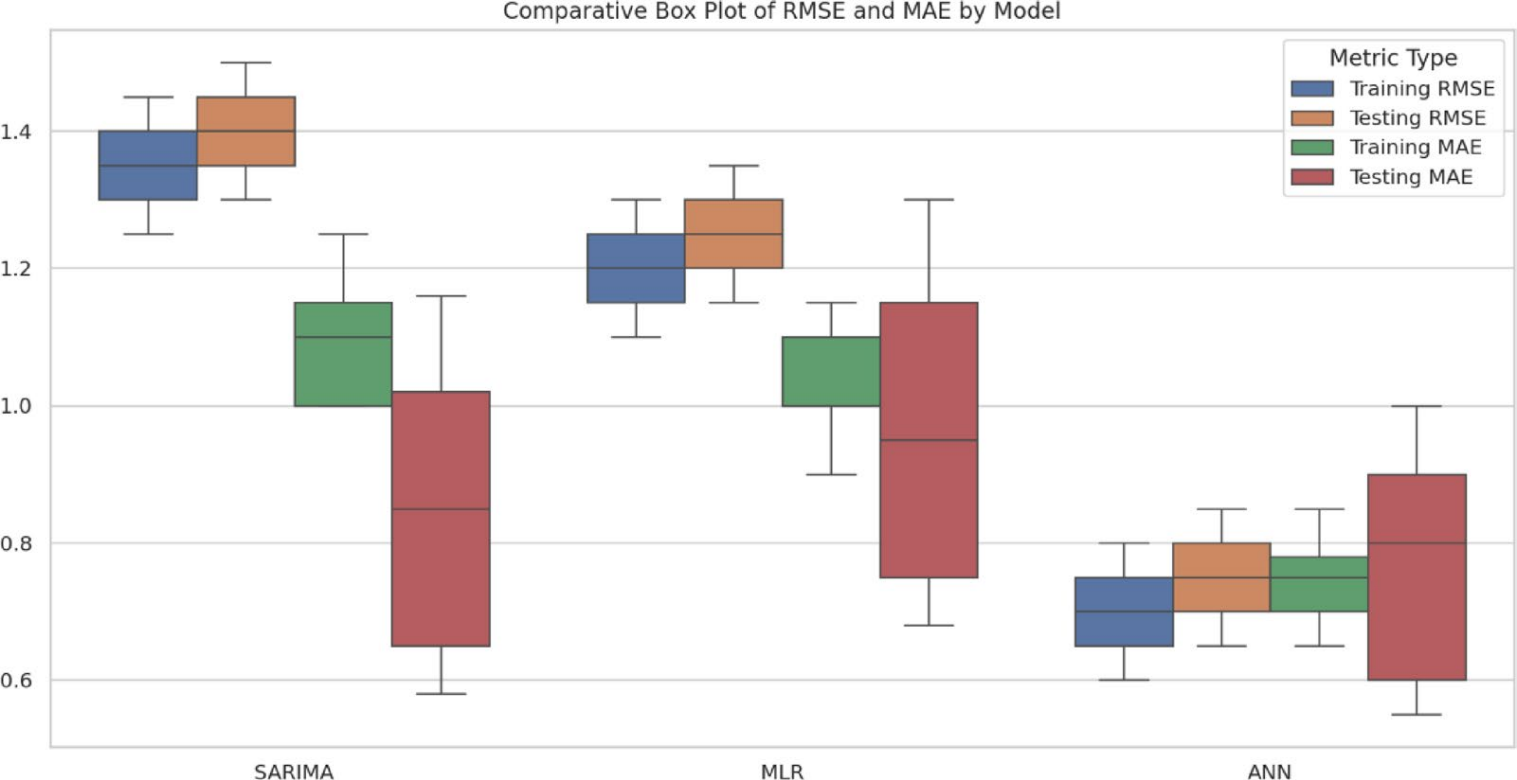
Comparative Box Plot of RMSE and MAE for all models.

**Fig. 12:**
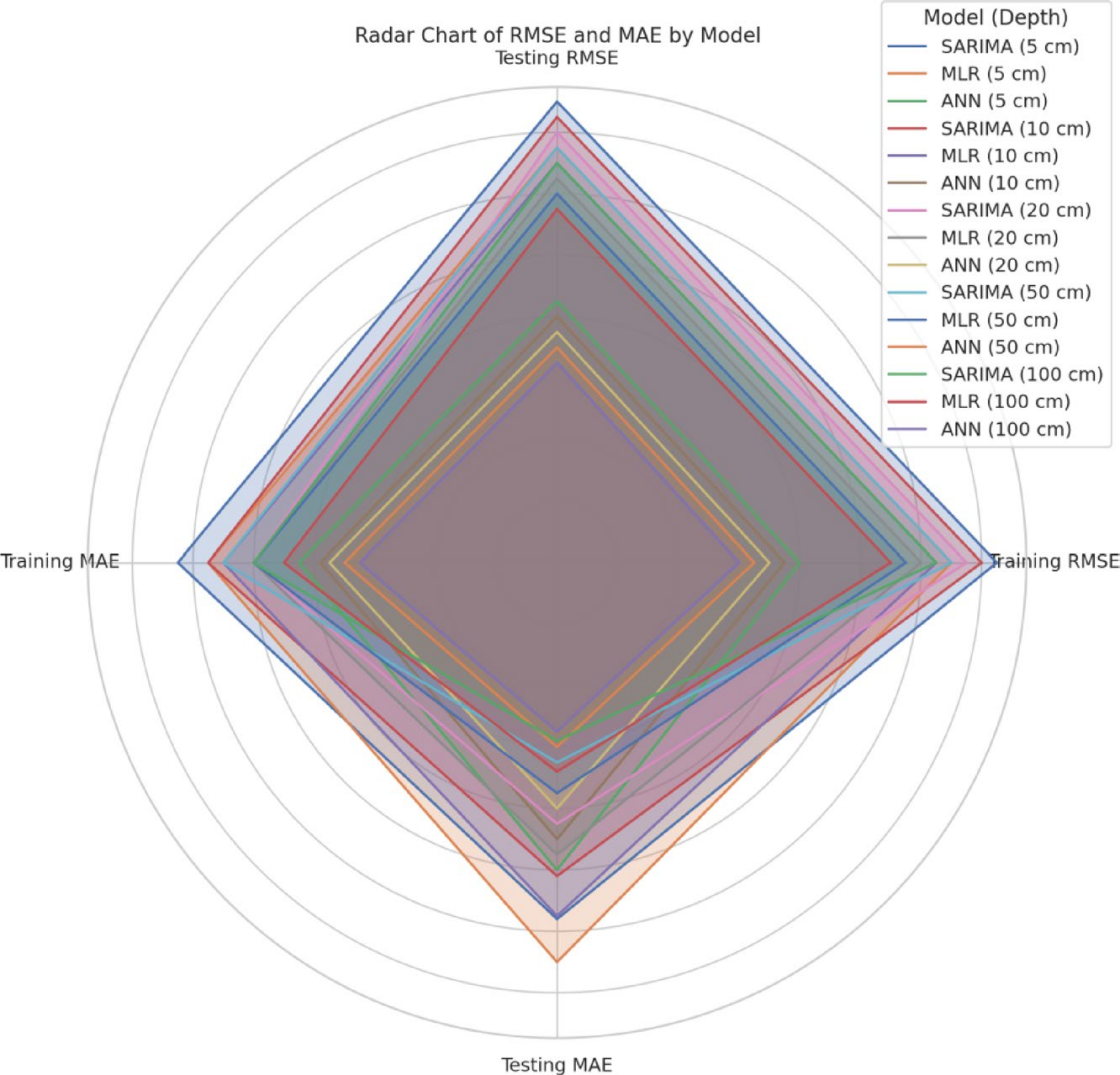
Comparative radar chart of RMSE and MAE for all models and soil depths.

Table 13 shows the performance comparison of implemented Models for Training and Testing Datasets. A comparative box plot and a radar chart (Figs. 11 and 12) are included to show the distribution of model predictions at different soil depths. These visualizations provide a more explicit representation of changes in model performance. ANN model offers the most accurate and reliable forecasts of daily ST, outperforming both the SARIMA and MLR models. This underscores the potential of ANN to capture complex, non-linear relationships between soil temperature and various meteorological parameters.

**Table 13:** Performance comparison of SARIMA, MLR, and ann models for training and testing datasets.

Depth (cm)	Model	Training RMSE	Training *r*	Training PBIAS (%)	Training MAE	Training *R*²	Testing RMSE	Testing *r*	Testing PBIAS (%)	Testing MAE	Testing *R*²
5	SARIMA	1.45	0.96	2.7	1.25	0.91	1.5	0.96	2.5	1.16	0.92
5	MLR	1.3	0.97	3.2	1.15	0.89	1.35	0.97	3	1.3	0.88
5	ANN	0.8	0.99	1.6	0.85	0.98	0.85	0.98	1.5	1	0.98
10	SARIMA	1.4	0.96	2.5	1.15	0.93	1.45	0.97	2.3	1.02	0.94
10	MLR	1.25	0.97	3	1.1	0.91	1.3	0.96	2.8	1.15	0.9
10	ANN	0.75	0.99	1.4	0.78	0.98	0.8	0.97	1.3	0.9	0.98
20	SARIMA	1.35	0.96	2	1	0.95	1.4	0.97	1.8	0.85	0.96
20	MLR	1.2	0.97	2.7	1	0.93	1.25	0.95	2.5	0.95	0.92
20	ANN	0.7	0.99	1.2	0.75	0.98	0.75	0.96	1	0.8	0.98
50	SARIMA	1.3	0.96	3.5	1.1	0.91	1.35	0.96	3.2	0.65	0.9
50	MLR	1.15	0.92	3.8	1	0.88	1.2	0.91	3.5	0.75	0.87
50	ANN	0.65	0.96	2.3	0.7	0.94	0.7	0.93	2	0.6	0.94
100	SARIMA	1.25	0.96	3.8	1	0.89	1.3	0.96	3.5	0.58	0.87
100	MLR	1.1	0.92	4	0.9	0.85	1.15	0.91	3.8	0.68	0.83
100	ANN	0.6	0.96	2.5	0.65	0.92	0.65	0.91	2.2	0.55	0.92

### Comparison with similar investigations

**Fig. 13:**
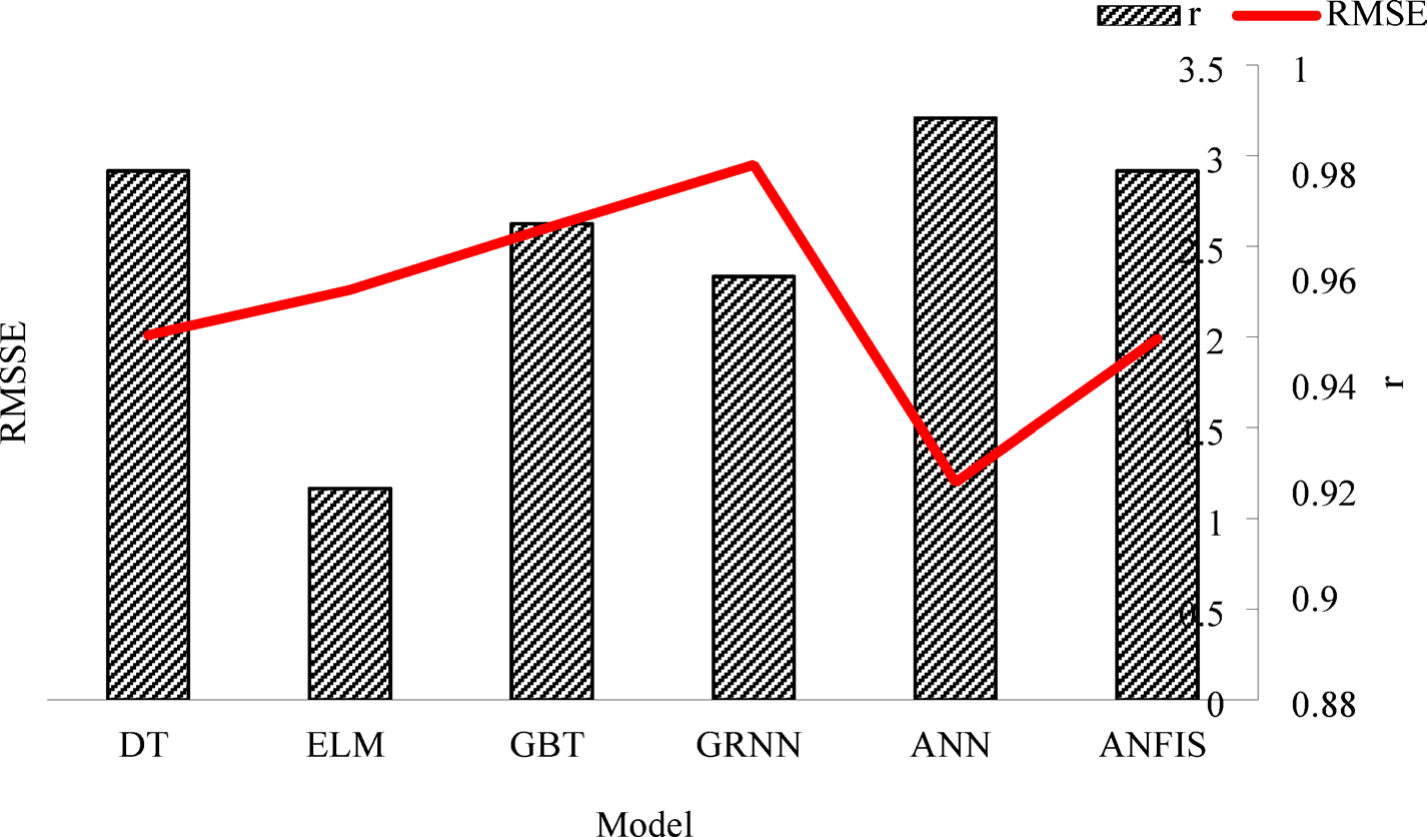
Comparison of intelligent prediction methods for soil temperature (ST) using RMSE and correlation coefficient (r). Data for ANFIS, Decision Tree (DT), Gradient Boosted Trees (GBT), Extreme Learning Machines (ELM), and Generalized Regression Neural Networks (GRNN) were sourced from previous studies^3,6,18^ and normalized for consistent evaluation with the ANN model developed in this study.

The statistical criteria values for five intelligent methods, including the ANFIS model^18^, decision tree (DT) and gradient boosted trees (GBT)^41^, ELM, and generalized regression neural networks (GRNN)^3^ are shown in Fig. 13. The data presented in Fig. 13 were sourced from prior studies that evaluated the performance of intelligent methods for soil temperature prediction under similar conditions. Performance metrics, including RMSE and r values, were extracted from the referenced literature^3,18,41^. These values were normalized where necessary to ensure consistency in evaluation criteria and enable a direct comparison with the present study. The metrics for the ANN model proposed in this study were calculated based on experimental results obtained using the same dataset and validation methods. This comparative analysis highlights the superior performance of the ANN model, particularly in achieving lower RMSE and higher correlation coefficients (r) compared to the alternative methods.

According to the evaluation criteria results, the ANN technique is within a more appropriate and acceptable error range for estimating daily ST than other intelligent methods. Furthermore, the ANN method has adequate power to distinguish between various daily ST levels. Compared to other methods, ANN performed well under nearly identical circumstances. Applying the proper parameters allows ANN to predict daily ST with reasonable accuracy. Although having more input parameters available yields more accurate solutions, intelligent models’ ability to adapt to input data makes it possible to pick the best available options while still getting the outcomes you want. ANFIS, DT, GBT, ELM, and GRNN all improved in determination coefficient criteria by 1–2%, respectively. The best method for estimating daily ST can be generally introduced as an intelligent strategies. The high accuracy of the ANN can lead to an accurate estimation of ST in different regions based on the data available in other stations. The advantage of the ANN is that its estimated results are close to the actual field conditions. The analysis considers ST levels at different soil depths in ANN. Also, ANN can provide an appropriate estimation of ST by only having soil temperature values in various soil layers, which reduces calculation time for moving applications. In general, it is suggested to work on the real-time applications of the ANN model and measure other soil conditions, such as the amount of salt and other elements present on its accuracy. The performance obtained by the ANN model is far from the ideal state, and there is room for more improvement in the field.

The findings of this study underline the critical importance of accurate soil temperature (ST) prediction for agricultural and environmental management. By addressing the limitations of direct ST measurements, such as high costs and limited synoptic station coverage, this research demonstrates the efficacy of data-driven models in filling these gaps.

Among the models evaluated, the Artificial Neural Network (ANN) model stands out for its superior performance in predicting ST at multiple depths. The ANN’s ability to capture complex non-linear interactions between meteorological parameters and ST highlights its robustness and adaptability compared to traditional methods like Multiple Linear Regression (MLR) and Seasonal ARIMA (SARIMA). This study’s application of ANN to soil temperature modeling represents a significant advancement, notably as it demonstrates the model’s ability to generalize across varying soil depths with minimal error, as evidenced by its high correlation coefficient (*r* = 0.98) and low RMSE values.

The findings also highlight the depth-specific influence of meteorological parameters on ST, with surface temperature (Avg. Infrared) playing a dominant role at shallow depths (5, 10, and 20 cm), while air temperature (Avg. T) becomes the key predictor at deeper levels (50 and 100 cm). These insights provide a deeper understanding of the thermal dynamics within soil profiles, which is crucial for optimizing agricultural practices such as irrigation scheduling, crop management, and climate-resilient farming.

Furthermore, the comparative analysis with other intelligent prediction methods, such as ANFIS, Decision Trees, and gradient-boosted trees, underscores the ANN model’s ability to outperform these alternatives under similar conditions. This comparative framework validates the methodological approach adopted in this study and provides a benchmark for future research in soil temperature modeling.

In summary, this research substantially contributes to the field by offering a scalable, efficient, and accurate framework for soil temperature prediction. Integrating advanced machine learning techniques with real-world meteorological data bridges the gap between theoretical advancements and practical applications, providing actionable insights for sustainable agricultural and environmental management.

### Advantages and limitations of the proposed model

The proposed ANN model has shown high prediction accuracy (*r* = 0.98 and low RMSE values) at different soil depths (5 cm, 10 cm, 20 cm, 50 cm and 100 cm) and can successfully capture the complex, non-linear relationships between soil temperature and meteorological parameters. This provides a significant advantage in an area where traditional models (e.g., MLR and SARIMA) are limited. The model has yielded practical results both near the surface and in deep soil layers, and its ability to predict soil temperature using only meteorological data offers a great advantage, especially in cases where measurements are costly and time-consuming. In addition, its easy adaptability to different geographical regions and climatic conditions allows the model to have a wide range of applications. However, the model has some limitations. The ANN model requires a large amount of high-quality data for training, and its performance may deteriorate if the data set is limited or noisy. The complex structure of the model and the long training process may increase the computational cost, especially in large data sets. In addition, the lower performance in deep soil layers (50 cm and 100 cm) compared to near-surface layers is due to the more complex temperature dynamics in these layers. The fact that the model is considered a “black box” and its internal workings are challenging to interpret may be a limitation for users, especially in agricultural applications. Finally, the model depends on meteorological data; if these data are missing or incorrect, the forecast performance may be negatively affected.

## Conclusion

The findings underscore the ability of ANN to efficiently handle the intricate, non-linear interactions between multiple meteorological factors and ST, which traditional models like MLR or SARIMA may fail to capture. With its superior capacity for data-driven modeling, the ANN can serve as a reliable tool for predicting daily ST across various depths. Such models offer greater accuracy and provide significant cost and time savings, especially when compared to conventional methods that require extensive ground-based measurements and data collection efforts.

In addition to providing valuable insights into soil temperature modeling, this research highlights the potential for data mining techniques to replace or supplement traditional methods of ST determination. Using readily available meteorological data to generate accurate ST predictions could be a transformative tool for agricultural management, particularly in precision farming, climate adaptation, and soil health monitoring.

The original contribution of this study is the comprehensive comparison of Seasonal ARIMA (SARIMA), Multiple Linear Regression (MLR) and Artificial Neural Networks (ANN) models to predict daily soil temperature at different soil depths (5 cm, 10 cm, 20 cm, 50 cm and 100 cm). The analysis results show that the ANN model has the lowest RMSE (0.60–0.85) and the highest correlation coefficient (*r* ≈ 0.98) values, especially at 5 cm and 10 cm depths, surface temperature (Avg. Infrared) stands out as the most critical parameter, while air temperature (Avg. T) becomes more decisive at 50 cm and 100 cm depths. SARIMA and MLR models were limited by higher RMSE (1.15–1.50) and lower correlation coefficient (*r* ≈ 0.91–0.97) values, especially in deeper layers. These findings reveal that the ANN model offers a more cost-effective and scalable solution for soil temperature estimation, especially in regions with limited synoptic stations, thanks to its ability to capture non-linear relationships. The study contributes significantly to the literature by proposing a practical model that can be used in areas such as agricultural management and climate adaptation.

In conclusion, this study provides strong evidence for using advanced machine learning techniques, particularly ANN, to improve soil temperature prediction accuracy. By leveraging meteorological data and enhancing predictive models, it is possible to move toward more sustainable, efficient, and cost-effective approaches for soil temperature monitoring in agricultural and environmental applications.

Future studies can explore several directions to enhance model performance and applicability. First, similar analyses can be conducted in different geographical regions and climatic conditions to test the generalization ability of the models. Additionally, advanced deep learning models, such as LSTM and CNN, may improve prediction accuracy. Incorporating soil properties, including soil moisture, soil type, and organic matter content, could also enhance the model’s predictive capability. Real-time data collection through IoT-based systems can enable the development of dynamic prediction models. Moreover, investigating the effects of climate change on soil temperature can provide valuable insights for agricultural and environmental planning. Expanding the scope of model comparison by including algorithms such as SVM and Random Forest may help identify the most suitable approach. Furthermore, associating soil temperature predictions with agricultural practices can offer practical guidance to farmers. Lastly, meta-heuristic algorithms, such as Genetic Algorithms, can be employed to optimize model performance.

## Data Availability

The datasets used and/or analysed during the current study available from the corresponding author on reasonable request.
